# Consensus meta-analysis of genome-wide association studies for Alzheimer’s disease and related dementias

**DOI:** 10.1038/s41588-026-02583-1

**Published:** 2026-06-03

**Authors:** EADB, Céline Bellenguez, Atahualpa Castillo Morales, Najaf Amin, Sven J. van der Lee, Manon Muntaner, Kayenat Parveen, Fahri Küçükali, Benjamin Grenier-Boley, Sami Heikkinen, Itziar de Rojas, Maria Carolina Dalmasso, Luca Kleineidam, Oliver Peters, Anja Schneider, Martin Dichgans, Dan Rujescu, Norbert Scherbaum, Jürgen Deckert, Steffi Riedel-Heller, Lucrezia Hausner, Laura Molina-Porcel, Emrah Düzel, Timo Grimmer, Jens Wiltfang, Stefanie Heilmann-Heimbach, Susanne Moebus, Matthias Schmid, Thomas Tegos, Nikolaos Scarmeas, Oriol Dols-Icardo, Fermin Moreno, Jordi Pérez-Tur, María J. Bullido, Pau Pastor, Raquel Sánchez-Valle, Victoria Álvarez, Mercè Boada, Pablo García-González, Raquel Puerta, Pablo Mir, Luis M. Real, Gerard Piñol-Ripoll, Jose María García-Alberca, Eloy Rodriguez-Rodriguez, Hilkka Soininen, Alexandre de Mendonça, Shima Mehrabian, Jakub Hort, Martin Vyhnalek, Nicolai Sandau, Jiao Luo, Jesper Qvist Thomassen, Yolande A. L. Pijnenburg, Wiesje van der Flier, Harro Seelaar, Inez Ramakers, Janne Papma, Marc Hulsman, Gert-Jan Biessels, Caroline Graff, Hakan Thonberg, Abbe Ullgren, Goran Papenberg, Vilmantas Giedraitis, Malin Löwenmark, Lena Kilander, Julie Williams, Peter Holmans, Julie Le Borgne, Sagnik Palmal, Aude Nicolas, Philippe Amouyel, Anne Boland, Jean-François Deleuze, Gael Nicolas, Carole Dufouil, Florence Pasquier, Olivier Hanon, Stéphanie Debette, Edna Grünblatt, Julius Popp, Daniela Galimberti, Beatrice Arosio, Patrizia Mecocci, Vincenzo Solfrizzi, Lucilla Parnetti, Alessio Squassina, Lucio Tremolizzo, Barbara Borroni, Michael Wagner, Benedetta Nacmias, Marco Spallazzi, Davide Seripa, Innocenzo Rainero, Antonio Daniele, Paola Bossù, Carlo Masullo, Giacomina Rossi, Frank Jessen, Henne Holstege, Karen Mather, M. Victoria Fernandez, Patrick G. Kehoe, Magda Tsolaki, Cornelia van Duijn, Ruth Frikke-Schmidt, Roberta Ghidoni, Pascual Sánchez-Juan, Kristel Sleegers, Martin Ingelsson, Mikko Hiltunen, Rebecca Sims, Ole Andreassen, Agustín Ruiz, Alfredo Ramirez, Jean-Charles Lambert, EADI, Céline Bellenguez, Benjamin Grenier-Boley, Philippe Amouyel, Carole Dufouil, Florence Pasquier, Olivier Hanon, Stéphanie Debette, Jean-Charles Lambert, Bonn, Luca Kleineidam, Anja Schneider, Steffi Riedel-Heller, Stefanie Heilmann-Heimbach, Susanne Moebus, Matthias Schmid, Julius Popp, Michael Wagner, Alfredo Ramirez, ADGC, Magda Tsolaki, Adam C. Naj, Farid Rajabli, Penelope Benchek, Lincoln M. P. Shade, Qi Qiao, Nicholas Kushch, Jin Sha, Katrina Bazemore, Congcong Zhu, Wan-Ping Lee, Jacob Haut, Kara L. Hamilton-Nelson, Nicholas R. Wheeler, Yi Zhao, John J. Farrell, Michelle A. Grunin, Yuk Yee Leung, Pavel P. Kuksa, Donghe Li, Eder Lucio da Fonseca, Jesse B. Mez, Ellen L. Palmer, Jagan Pillai, Richard M. Sherva, Yeunjoo E. Song, Xiaoling Zhang, Takeshi Ikeuchi, Taha Iqbal, Otto Valladares, Dolly Reyes-Dumeyer, Amanda B. Kuzma, Erin Abner, Larry D. Adams, Alyssa Aguirre, Marilyn S. Albert, Roger L. Albin, Mariet Allen, Liana G. Apostolova, Steven E. Arnold, Sanjay Asthana, Craig S. Atwood, Sanford Auerbach, Clinton T. Baldwin, Robert C. Barber, Lisa L. Barnes, Sandra Barral, Thomas G. Beach, James T. Becker, Gary W. Beecham, Duane Beekly, David A. Bennett, John Bertelson, Thomas D. Bird, Deborah Blacker, Bradley F. Boeve, James D. Bowen, Adam Boxer, James Brewer, Jeffrey M. Burns, Joseph D. Buxbaum, Nigel J. Cairns, Laura B. Cantwell, Chuanhai Cao, Christopher S. Carlson, Cynthia M. Carlsson, Regina M. Carney, Minerva M. Carrasquillo, Marie-Francoise Chesselet, Nathaniel A. Chin, Helena C. Chui, Jaeyoon Chung, Steven A. Claas, Suzanne Craft, Paul K. Crane, David H. Cribbs, Elizabeth A. Crocco, Carlos Cruchaga, Michael L. Cuccaro, Munro Cullum, Eveleen Darby, Barbara Davis, Philip L. De Jager, Charles DeCarli, John DeToledo, Malcolm Dick, Dennis W. Dickson, Beth A. Dombroski, Rachelle S. Doody, Ranjan Duara, Logan C. Dumitrescu, Nilüfer Ertekin-Taner, Denis A. Evans, Kelley M. Faber, Thomas J. Fairchild, Kenneth B. Fallon, Martin R. Farlow, Victoria Fernandez-Hernandez, Robert P. Friedland, Tatiana M. Foroud, Matthew P. Frosch, Brian Fulton-Howard, Douglas R. Galasko, Marla Gearing, Daniel H. Geschwind, Bernardino Ghetti, John R. Gilbert, Rodney C. P. Go, Alison M. Goate, Thomas J. Grabowski, Neill R. Graff-Radford, Nora E. Gray, John H. Growdon, Hakon Hakonarson, James Hall, Ronald L. Hamilton, Oscar Harari, John Hardy, Elizabeth Head, Victor W. Henderson, Michelle Hernandez, Timothy J. Hohman, Lawrence S. Honig, Ryan M. Huebinger, Matthew J. Huentelman, Bradley T. Hyman, Linda S. Hynan, Laura Ibanez, Gail P. Jarvik, Suman Jayadev, Lee-Way Jin, Kim Johnson, Leigh Johnson, M. Ilyas Kamboh, Yuriko Katsumata, Mindy J. Katz, John S. Kauwe, Jeffrey A. Kaye, C. Dirk Keene, Aisha Khaleeq, Masataka Kikuchi, Janice Knebl, Neil W. Kowall, Joel H. Kramer, Walter A. Kukull, Frank M. LaFerla, James J. Lah, Eric B. Larson, Alan Lerner, James B. Leverenz, Allan I. Levey, Andrew P. Lieberman, Richard B. Lipton, Mark Logue, Oscar L. Lopez, Kathryn L. Lunetta, Constantine G. Lyketsos, Douglas Mains, Daniel C. Marson, Eden R. R. Martin, Frank Martiniuk, Deborah C. Mash, Eliezer Masliah, Paul Massman, Arjun Masurkar, Wayne C. McCormick, Susan M. McCurry, Ann C. McKee, Marsel Mesulam, Bruce L. Miller, Carol A. Miller, Joshua W. Miller, Thomas J. Montine, Edwin S. Monuki, John C. Morris, Shubhabrata Mukherjee, Amanda J. Myers, Trung Nguyen, Thomas Obisesan, Sid O’Bryant, John M. Olichney, Raymond Palmer, Joseph E. Parisi, Henry L. Paulson, Valory Pavlik, David Paydarfar, Victoria Perez, Elaine Peskind, Ronald C. Petersen, Helen Petrovitch, Marsha Polk, Wayne W. Poon, Huntington Potter, Liming Qu, Mary Quiceno, Joseph F. Quinn, Ashok Raj, Murray Raskind, Eric M. Reiman, Barry Reisberg, Joan S. Reisch, John M. Ringman, Erik D. Roberson, Monica Rodriguear, Ekaterina Rogaeva, Howard J. Rosen, Roger N. Rosenberg, Donald R. Royall, Marwan Sabbagh, A. Dessa Sadovnick, Mark A. Sager, Mary Sano, Andrew J. Saykin, Julie A. Schneider, Lon S. Schneider, William W. Seeley, Susan H. Slifer, Scott Small, Amanda G. Smith, Joshua A. Sonnen, Peter St George-Hyslop, Takiyah D. Starks, Robert A. Stern, Alan B. Stevens, Stephen M. Strittmatter, David Sultzer, Russell H. Swerdlow, Rudolph E. Tanzi, Jeffrey L. Tilson, Juan C. Troncoso, Debby W. Tsuang, Vivianna M. Van Deerlin, Linda J. Van Eldik, Jeffery M. Vance, Badri N. Vardarajan, Robert Vassar, Harry V. Vinters, Jean-Paul Vonsattel, Sandra Weintraub, Kathleen A. Welsh-Bohmer, Patrice L. Whitehead, Ellen M. Wijsman, Kirk C. Wilhelmsen, Benjamin Williams, Jennifer Williamson, Henrik Wilms, Thomas S. Wingo, Thomas Wisniewski, Randall L. Woltjer, Clinton B. Wright, Chuang-Kuo Wu, Steven G. Younkin, Chang-En Yu, Lei Yu, Xiongwei Zhu, Brian W. Kunkle, William S. Bush, Akinori Miyashita, Giuseppe Tosto, Gyungah R. Jun, Christiane Reitz, Goldie S. Byrd, David W. Fardo, Li-San Wang, Lindsay A. Farrer, Jonathan L. Haines, Richard Mayeux, Margaret A. Pericak-Vance, Gerard D. Schellenberg, CHARGE, Carole Dufouil, Stéphanie Debette, Bernard Fongang, Amber Yaqub, Muralidharan Sargurupremraj, Xueqiu Jian, Aniket Mishra, Joshua C. Bis, Monica Gireud-Goss, Jayandra Jung Himali, Habil Zare, Vilmundur Guðnason, Lenore Launer, Jan Bressler, Hans J. Grabe, M. Arfan Ikram, Bruce M. Psaty, W. T. Longstreth, Sigurdur Sigurdsson, Mohsen Ghanbari, Franck J. Wolters, Eric Boerwinkle, Alexa S. Beiser, Chloe Sarnowski, Thomas H. Mosley, Oscar L. Lopez, Cornelia van Duijn, Claudia Satizabal, M. Kamran Ikram, Yang Qiong, Myriam Fornage, Sudha Seshadri, FinnGen, Sami Heikkinen, Hilkka Soininen, Mikko Hiltunen, GERAD, Atahualpa Castillo Morales, Julie Williams, Peter Holmans, Patrick G. Kehoe, Rebecca Sims, Rebecca Mahoney, Nicola Denning, Alun Meggy, Rachel Marshall, Danielle LeRoux, Catherine Bresner, Valentina Escott-Price, Kevin Morgan, Keeley Brookes, Tamar Guetta-Baranes, Clive Holmes, Gill Windle, Vanessa Burholt, Emma Green, Catherine Macleod, Bob Woods, Simon Mead, Jonathan M. Schott, Nick Fox, Seth Love, GR@ACE/DEGESCO, Itziar de Rojas, Laura Molina-Porcel, Oriol Dols-Icardo, Fermin Moreno, Jordi Pérez-Tur, María J. Bullido, Pau Pastor, Raquel Sánchez-Valle, Victoria Álvarez, Mercè Boada, Pablo García-González, Raquel Puerta, Pablo Mir, Gerard Piñol-Ripoll, Jose María García-Alberca, Eloy Rodriguez-Rodriguez, Pascual Sánchez-Juan, Agustín Ruiz, Clàudia Olivé, Laura Montrreal, M. Victoria Fernández, Marta Marquié, Amanda Cano, Sergi Valero, Oscar Sotolongo-Grau, Alba Pérez-Cordón, Ana Espinosa, Ángela Sanabria, Gemma Ortega, Maitée Rosende-Roca, Montserrat Alegret, Lluís Tárraga, María Eugenia Sáez, Inés Quintela, Ángel Carracedo, Luis M. Real, Juan Macías, Anaïs Corma-Gómez, Juan A. Pineda, Silvia Mendoza, Jose Luis Royo, Guillermo Garcia-Ribas, Sebastián García-Madrona, Emilio Franco-Macías, Dolores Buiza-Rueda, María Bernal Sánchez-Arjona, Raquel Huerto Vilas, Alfonso Arias Pastor, Mónica Diez-Fairen, Ignacio Alvarez, Carmen Lage, Daniel Alcolea, Juan Fortea, Alberto Lleó, Ana Frank-García, Angel Martín Montes, Anna Antonell, Manuel Menéndez-González, Adolfo Lopez de Munain, Miguel Medina, Miguel Calero, Alberto Rábano, Ana Belén Pastor, Teodoro del Ser, Florentino Sanchez-Garcia, Carmen Muñoz-Fernandez, M. Candida Deniz-Naranjo, PGC-ALZ, Danielle Posthuma, Ole A. Andreassen, Douglas P. Wightman, Emil Uffelmann, Hreinn Stefansson, G. Bragi Walters, Kari Stefansson, Jon Snaedal, Helga Eyjólfsdóttir, Nancy L. Pedersen, Chandra A. Reynolds, Ida K. Karlsson, Sara Hägg, Anna Zettergren, Ingmar Skoog, Silke Kern, Margda Waern, Kaj Blennow, Henrik Zetterberg, Elisa Moreno, Marta Riise Moksnes, Kristian Hveem, Bendik S. Winsvold, Ben Brumpton, Geir Selbæk, Tormod Fladby, Dag Aarsland, Srdjan Djurovic, Arvid Rongve, Shahram Bahrami, Alexey A. Shadrin, Ingvild Saltvedt, Geir Bråthen

**Affiliations:** 1 Univ. Lille, Inserm, CHU Lille, Institut Pasteur Lille, LabEx DISTALZ—U1167-RID-AGE Facteurs de Risque et Déterminants Moléculaires des Maladies Liées au Vieillissement, Lille, France; 2 UKDRI@ Cardiff, School of Medicine, Cardiff University, Cardiff, UK; 3 Nuffield Department of Population Health, University of Oxford, Oxford, UK; 4 Alzheimer Center Amsterdam, Neurology, Vrije Universiteit Amsterdam, Amsterdam UMC location VUmc, Amsterdam, The Netherlands; 5 Department of Complex Trait Genetics, Center for Neurogenomics and Cognitive Research, Amsterdam Neuroscience, Vrije University, Amsterdam, The Netherlands; 6 Amsterdam Neuroscience, Neurodegeneration, Amsterdam, The Netherlands; 7 Division of Neurogenetics and Molecular Psychiatry, Department of Psychiatry and Psychotherapy, Faculty of Medicine and University Hospital Cologne, University of Cologne, Cologne, Germany; 8 Department of Cognitive Disorders and Old Age Psychiatry, University Hospital Bonn, Medical Faculty, Bonn, Germany; 9 Complex Genetics of Alzheimer’s Disease Group, VIB Center for Molecular Neurology, VIB, Antwerp, Belgium; 10 Department of Biomedical Sciences, University of Antwerp, Antwerp, Belgium; 11 Institute of Biomedicine, Faculty of Health Sciences, University of Eastern Finland, Kuopio, Finland; 12 Ace Alzheimer Center Barcelona, Universitat Internacional de Catalunya (UIC), Barcelona, Spain; 13 Networking Research Center on Neurodegenerative Diseases (CIBERNED), Instituto de Salud Carlos III, Madrid, Spain; 14 Estudios en Neurociencias y Sistemas Complejos (ENyS) CONICET-HEC-UNAJ, Florencio Varela, Argentina; 15 Department of Neurodegeneration and Geriatric Psychiatry, University of Bonn, Bonn, Germany; 16 German Center for Neurodegenerative Diseases (DZNE Bonn), Bonn, Germany; 17 German Center for Neurodegenerative Diseases (DZNE), Berlin, Germany; 18 Institute of Psychiatry and Psychotherapy, Charité—Universitätsmedizin Berlin, Corporate member of Freie Universität Berlin, Humboldt Universität zu Berlin and Berlin Institute of Health, Berlin, Germany; 19 German Center for Neurodegenerative Diseases (DZNE), Bonn, Germany; 20 Department of Cognitive Disorders and Old Age Psychiatry, University Hospital Bonn, Venusberg-Campus 1, Bonn, Germany; 21 Institute for Stroke and Dementia Research (ISD), University Hospital, LMU Munich, Munich, Germany; 22 German Center for Neurodegenerative Diseases (DZNE), Munich, Germany; 23 Munich Cluster for Systems Neurology (SyNergy), Munich, Germany; 24 Martin-Luther-University Halle-Wittenberg, University Clinic and Outpatient Clinic for Psychiatry, Psychotherapy and Psychosomatics, Halle (Saale), Germany; 25 LVR-University Hospital Essen, Department of Psychiatry and Psychotherapy, Medical Faculty, University of Duisburg-Essen, Essen, Germany; 26 Department of Psychiatry, Psychosomatics and Psychotherapy, Center of Mental Health, University Hospital of Würzburg, Würzburg, Germany; 27 Institute of Social Medicine, Occupational Health and Public Health, University of Leipzig, Leipzig, Germany; 28 Department of Geriatric Psychiatry, Central Institute for Mental Health Mannheim, Faculty Mannheim, University of Heidelberg, Mannheim, Germany; 29 Alzheimer’s Disease and Other Cognitive Disorders Unit, Service of Neurology, Hospital Clínic of Barcelona, Institut d’Investigacions Biomèdiques August Pi i Sunyer, Institute of Neurosciences of the University of Barcelona, Barcelona, Spain; 30 Neurological Tissue Bank - Biobanc - Hospital Clínic - IDIBAPS, Barcelona, Spain; 31 German Center for Neurodegenerative Diseases (DZNE), Magdeburg, Germany; 32 Institute of Cognitive Neurology and Dementia Research (IKND), Otto-von-Guericke University, Magdeburg, Germany; 33 Center for Cognitive Disorders, Department of Psychiatry and Psychotherapy, Technical University of Munich, School of Medicine, Munich, Germany; 34 Department of Psychiatry and Psychotherapy, University Medical Center Goettingen, Goettingen, Germany; 35 German Center for Neurodegenerative Diseases (DZNE), Goettingen, Germany; 36 Medical Science Department, iBiMED, Aveiro, Portugal; 37 Institute of Human Genetics, University of Bonn, School of Medicine & University Hospital Bonn, Bonn, Germany; 38 Institute for Urban Public Health, University Hospital of University Duisburg-Essen, Essen, Germany; 39 German Center for Neurodegenerative Diseases (DZNE, Bonn), Bonn, Germany; 40 Institute of Medical Biometry, Informatics and Epidemiology, University Hospital of Bonn, Bonn, Germany; 41 1st Department of Neurology, Medical school, Aristotle University of Thessaloniki, Thessaloniki, Makedonia Greece; 42 Laboratory of Neurodegenerative Diseases, Center for Interdisciplinary Research and Innovation (CIRI - AUTh), Balkan Center, Aristotle University of Thessaloniki, Thessaloniki, Greece; 43 Greek Alzheimer Disease Association and Related Disorders, Thessaloniki, Greece; 44 Taub Institute for Research in Alzheimer’s Disease and the Aging Brain, The Gertrude H. Sergievsky Center, Department of Neurology, Columbia University, New York, NY USA; 45 1st Department of Neurology, Aiginition Hospital, National and Kapodistrian University of Athens, Medical School, Athens, Greece; 46 Sant Pau Memory Unit, Institut de Recerca Sant Pau, Department of Neurology, Hospital de la Santa Creu i Sant Pau, Universitat Autònoma de Barcelona, Barcelona, Spain; 47 Department of Neurology, Hospital Universitario Donostia, San Sebastian, Spain; 48 Neurosciences Area, Instituto Biodonostia, San Sebastian, Spain; 49 Unitat de Genètica Molecular, Institut de Biomedicina de València-CSIC, Valencia, Spain; 50 Centro de Biología Molecular Severo Ochoa (UAM-CSIC), Madrid, Spain; 51 Instituto de Investigacion Sanitaria ‘Hospital la Paz’ (IdIPaz), Madrid, Spain; 52 Universidad Autónoma de Madrid, Madrid, Spain; 53 Unit of Neurodegenerative diseases, Department of Neurology, Hospital Germans Trias i Pujol, Badalona, Barcelona, Spain; 54 Neurociences, Germans Trias i Pujol Research Institute (IGTP), Badalona, Barcelona, Spain; 55 Alzheimer’s disease and other cognitive disorders unit, Service of Neurology, Hospital Clínic of Barcelona, Institut d’Investigacions Biomèdiques August Pi i Sunyer, University of Barcelona, Barcelona, Spain; 56 Laboratorio de Genética, Hospital Universitario Central de Asturias, Oviedo, Spain; 57 Instituto de Investigación Sanitaria del Principado de Asturias (ISPA), Oviedo, Spain; 58 Unidad de Trastornos del Movimiento, Servicio de Neurología y Neurofisiología, Instituto de Biomedicina de Sevilla (IBiS), Hospital Universitario Virgen del Rocío/CSIC/Universidad de Sevilla, Seville, Spain; 59 Unidad Clínica de Enfermedades Infecciosas y Microbiología. Hospital Universitario de Valme, Sevilla, Spain; 60 Depatamento de Especialidades Quirúrgicas, Bioquímica e Inmunología, Facultad de Medicina, Universidad de Málaga, Málaga, Spain; 61 Unitat Trastorns Cognitius, Hospital Universitari Santa Maria de Lleida, Lleida, Spain; 62 Institut de Recerca Biomedica de Lleida (IRBLLeida), Lleida, Spain; 63 Alzheimer Research Center & Memory Clinic, Andalusian Institute for Neuroscience, Málaga, Spain; 64 Neurology Service, Marqués de Valdecilla University Hospital (University of Cantabria and IDIVAL), Santander, Spain; 65 Department of Neurology, Institute of Clinical Medicine, University of Eastern Finland, Kuopio, Finland; 66 Faculty of Medicine, University of Lisbon, Lisbon, Portugal; 67 Clinic of Neurology, UH “Alexandrovska”, Medical University - Sofia, Sofia, Bulgaria; 68 Memory Clinic, Department of Neurology, Charles University, Second Faculty of Medicine and Motol University Hospital, Prague, Czech Republic; 69 International Clinical Research Center, St. Anne’s University Hospital Brno, Brno, Czech Republic; 70 Department of Clinical Biochemistry, Copenhagen University Hospital - Rigshospitalet, Copenhagen, Denmark; 71 Maastricht University, Department of Psychiatry & Neuropsychologie, Alzheimer Center Limburg, Maastricht, The Netherlands; 72 Karolinska Institutet, Center for Alzheimer Research, Department NVS, Division of Neurogeriatrics, Stockholm, Sweden; 73 Unit for Hereditary Dementias, Karolinska University Hospital-Solna, Stockholm, Sweden; 74 Aging Research Center, Department of Neurobiology, Care Sciences and Society, Karolinska Institutet and Stockholm University, Stockholm, Sweden; 75 Department of Public Health and Caring Sciences, Molecular Geriatrics, Rudbeck Laboratory, Uppsala University, Uppsala, Sweden; 76 Centre for Neuropsychiatric Genetics and Genomics, Division of Psychological Medicine and Clinical Neuroscience, School of Medicine, Cardiff University, Cardiff, UK; 77 Moondance Dementia Research Laboratory, Cardiff, UK; 78 Université Paris-Saclay, CEA, Centre National de Recherche en Génomique Humaine, Evry, France; 79 University Rouen Normandie, Normandie University, Inserm U1245 and Department of Genetics and CNRMAJ, CHU Rouen, Rouen, France; 80 Inserm, Bordeaux Population Health Research Center, UMR 1219, ISPED, CIC 1401-EC, Univ. Bordeaux, Bordeaux, France; 81 CHU de Bordeaux, Pole santé publique, Bordeaux, France; 82 Inserm 1171, CHU Clinical and Research Memory Research Centre (CMRR) of DISTALZ, Univ. Lille, Lille, France; 83 Université de Paris, EA 4468, APHP, Hôpital Broca, Paris, France; 84 University Bordeaux, Inserm, Bordeaux Population Health Research Center, Bordeaux, France; 85 Department of Neurology, Bordeaux University Hospital, Bordeaux, France; 86 Department of Child and Adolescent Psychiatry and Psychotherapy, University Hospital of Psychiatry Zurich, University of Zurich, Zurich, Switzerland; 87 Neuroscience Center Zurich, University of Zurich and ETH Zurich, Zurich, Switzerland; 88 Zurich Center for Integrative Human Physiology, University of Zurich, Zurich, Switzerland; 89 Old Age Psychiatry, Department of Psychiatry, Lausanne University Hospital, Lausanne, Switzerland; 90 Department of Geriatric Psychiatry, University Hospital of Psychiatry Zürich, Zürich, Switzerland; 91 Institute for Regenerative Medicine, University of Zürich, Zürich, Switzerland; 92 Neurodegenerative Diseases Unit, Fondazione IRCCS Ca’ Granda, Ospedale Policlinico, Milan, Italy; 93 Dept. of Biomedical, Surgical and Dental Sciences, University of Milan, Milan, Italy; 94 Department of Clinical Sciences and Community Health, University of Milan, Milan, Italy; 95 Geriatric Unit, Fondazione IRCCS Ca’ Granda Ospedale Maggiore Policlinico, Milan, Italy; 96 Institute of Gerontology and Geriatrics, Department of Medicine and Surgery, University of Perugia, Perugia, Italy; 97 Division of Clinical Geriatrics, Department of Neurobiology, Care Sciences and Society, Karolinska Institutet, Stockholm, Sweden; 98 Interdisciplinary Department of Medicine, Geriatric Medicine and Memory Unit, University of Bari “A. Moro, Bari, Italy; 99 Academic Division “C. Frugoni” & Hospital Division of Internal and Geriatric Medicine, Policlinico Hospital, Bari, Italy; 100 Centre for Memory Disturbances, Lab of Clinical Neurochemistry, Section of Neurology, University of Perugia, Perugia, Italy; 101 Department of Biomedical Sciences, Section of Neuroscience and Clinical Pharmacology, University of Cagliari, Cagliari, Italy; 102 Neurology Unit, IRCCS “San Gerardo dei Tintori”, Monza, Italy; 103 School of Medicine and Surgery, University of Milano-Bicocca, Monza, Italy; 104 Department of Clinical and Experimental Sciences, University of Brescia, Brescia, Italy; 105 Molecular Markers Laboratory, IRCCS Istituto Centro San Giovanni di Dio Fatebenefratelli, Brescia, Italy; 106 Department of Neuroscience, Psychology, Drug Research and Child Health University of Florence, Florence, Italy; 107 IRCCS Fondazione Don Carlo Gnocchi, Florence, Italy; 108 Department of Medicine and Surgery, Unit of Neurology, University-Hospital of Parma, Parma, Italy; 109 Department of Hematology and Stem Cell Transplant, Vito Fazzi Hospital, Lecce, Italy; 110 Department of Neuroscience “Rita Levi Montalcini”, University of Torino, Torino, Italy; 111 Department of Neuroscience, Università Cattolica del Sacro Cuore, Rome, Italy; 112 Neurology Unit, IRCCS Fondazione Policlinico Universitario A. Gemelli, Rome, Italy; 113 Laboratory of Experimental Neuropsychobiology, Clinical Neuroscience and Neurorehabilitation Department, IRCCS Santa Lucia Foundation, Rome, Italy; 114 Institute of Neurology, Catholic University of the Sacred Heart, Rome, Italy; 115 Unit of Neurology V - Neuropathology, Fondazione IRCCS Istituto Neurologico Carlo Besta, Milan, Italy; 116 Department of Psychiatry and Psychotherapy, Faculty of Medicine and University Hospital Cologne, University of Cologne, Cologne, Germany; 117 Cluster of Excellence Cellular Stress Responses in Aging-associated Diseases (CECAD), University of Cologne, Cologne, Germany; 118 Genomics of Neurodegenerative Diseases and Aging, Human Genetics, Vrije Universiteit Amsterdam, Amsterdam UMC location VUmc, Amsterdam, The Netherlands; 119 Centre for Healthy Brain Ageing, Discipline of Psychiatry and Mental Health, School of Clinical Medicine, Faculty of Medicine and Health, University of New South Wales, Sydney, New South Wales Australia; 120 Translational Health Sciences, Bristol Medical School, University of Bristol, Bristol, UK; 121 Department of Epidemiology, ErasmusMC, Rotterdam, The Netherlands; 122 Centre for Artificial Intelligence in Precision Medicine, University of Oxford, Oxford, UK; 123 Department of Clinical Medicine, University of Copenhagen, Copenhagen, Denmark; 124 Reina Sofia Alzheimer Center, CIEN Foundation, ISCIII, Madrid, Spain; 125 Krembil Brain Institute, University Health Network, Toronto, Ontario Canada; 126 Tanz Centre for Research in Neurodegenerative Diseases, Departments of Medicine and Laboratory Medicine & Pathobiology, University of Toronto, Toronto, Ontario, Canada; 127 NORMENT Centre, Division of Mental Health and Addiction, Oslo University Hospital, Oslo, Norway; 128 Institute of Clinical Medicine, University of Oslo, Oslo, Norway; 129 Biggs Institute for Alzheimer’s and Neurodegenerative Diseases, University of Texas Health Science Center, San Antonio, TX USA; 130 Department of Psychiatry & Glenn Biggs Institute for Alzheimer’s and Neurodegenerative Diseases, San Antonio, TX USA; 131 Cologne Excellence Cluster on Cellular Stress Responses in Aging-Associated Disease (CECAD), University of Cologne, Cologne, Germany; 132 Department of Biostatistics, Epidemiology, and Informatics, Perelman School of Medicine, University of Pennsylvania, Philadelphia, PA USA; 133 Penn Neurodegeneration Genomics Center, Department of Pathology and Laboratory Medicine, Perelman School of Medicine, University of Pennsylvania, Philadelphia, PA USA; 134 Dr. John T. Macdonald Foundation Department of Human Genetics, Miller School of Medicine, University of Miami, Miami, FL USA; 135 The John P. Hussman Institute for Human Genomics, University of Miami, Miami, FL USA; 136 Department of Population and Quantitative Health Sciences, School of Medicine, Case Western Reserve University, Cleveland, OH USA; 137 Department of Biostatistics, College of Public Health, University of Kentucky, Lexington, KY USA; 138 Sanders-Brown Center on Aging, University of Kentucky, Lexington, KY USA; 139 Department of Medicine (Biomedical Genetics), Boston University Chobanian & Avedisian School of Medicine, Boston, MA USA; 140 Cleveland Institute for Computational Biology, School of Medicine, Case Western Reserve University, Cleveland, OH USA; 141 Department of Neurology, Boston University Chobanian & Avedisian School of Medicine, Boston, MA USA; 142 Cleveland Clinic Lou Ruvo Center for Brain Health, Cleveland Clinic, Cleveland, OH USA; 143 Department of Biostatistics, Boston University School of Public Health, Boston, MA USA; 144 Molecular Genetics Division, Brain Research Institute, Niigata University, Niigata, Japan; 145 Department of Neurology, Columbia University, New York, NY USA; 146 Department of Epidemiology and Environmental Health, College of Public Health, University of Kentucky, Lexington, KY USA; 147 Department of Neurology, Dell Medical School, University of Texas at Austin, Austin, TX USA; 148 Department of Neurology, Johns Hopkins University, Baltimore, MD USA; 149 Department of Neurology, University of Michigan, Ann Arbor, MI USA; 150 Geriatric Research, Education and Clinical Center (GRECC), VA Ann Arbor Healthcare System (VAAAHS), Ann Arbor, MI USA; 151 Michigan Alzheimer’s Disease Center, University of Michigan, Ann Arbor, MI USA; 152 Department of Neuroscience, Mayo Clinic, Jacksonville, FL USA; 153 Departments of Neurology, Radiology, and Medical and Molecular Genetics, Indiana University School of Medicine, Indianapolis, IN USA; 154 Indiana Alzheimer’s Disease Research Center, Indiana University School of Medicine, Indianapolis, IN USA; 155 Department of Psychiatry, Perelman School of Medicine, University of Pennsylvania, Philadelphia, PA USA; 156 Geriatric Research, Education and Clinical Center (GRECC), University of Wisconsin, Madison, WI USA; 157 Department of Medicine, University of Wisconsin, Madison, WI USA; 158 Wisconsin Alzheimer’s Disease Research Center, Madison, WI USA; 159 Department of Pharmacology and Neuroscience, University of North Texas Health Science Center, Fort Worth, TX USA; 160 Department of Neurological Sciences, Rush University Medical Center, Chicago, IL USA; 161 Department of Behavioral Sciences, Rush University Medical Center, Chicago, IL USA; 162 Rush Alzheimer’s Disease Center, Rush University Medical Center, Chicago, IL USA; 163 Gertrude H. Sergievsky Center, Columbia University, New York, NY USA; 164 Civin Laboratory for Neuropathology, Banner Sun Health Research Institute, Phoenix, AZ USA; 165 Departments of Psychiatry, Neurology, and Psychology, University of Pittsburgh School of Medicine, Pittsburgh, PA USA; 166 National Alzheimer’s Coordinating Center, University of Washington, Seattle, WA USA; 167 Department of Psychiatry, University of Texas at Austin/Dell Medical School, Austin, TX USA; 168 VA Puget Sound Health Care System/GRECC, Seattle, WA USA; 169 Department of Neurology, University of Washington, Seattle, WA USA; 170 Department of Epidemiology, Harvard School of Public Health, Boston, MA USA; 171 Department of Psychiatry, Massachusetts General Hospital/Harvard Medical School, Boston, MA USA; 172 Department of Neurology, Mayo Clinic, Rochester, MN USA; 173 Swedish Medical Center, Seattle, WA USA; 174 Department of Neurology, University of California San Francisco, San Francisco, CA USA; 175 Department of Neurosciences, University of California San Diego, La Jolla, CA USA; 176 University of Kansas Alzheimer’s Disease Center, University of Kansas Medical Center, Kansas City, KS USA; 177 Department of Genetics and Genomic Sciences, Ronald M. Loeb Center for Alzheimer’s Disease, Icahn School of Medicine at Mount Sinai, New York, NY USA; 178 Department of Neuroscience, Icahn School of Medicine at Mount Sinai, New York, NY USA; 179 Department of Psychiatry, Mount Sinai School of Medicine, New York, NY USA; 180 Department of Pathology and Immunology, Washington University, St. Louis, MO USA; 181 USF Health Byrd Alzheimer’s Institute, University of South Florida, Tampa, FL USA; 182 Fred Hutchinson Cancer Research Center, Seattle, WA USA; 183 Mental Health and Behavioral Science Service, Bruce W. Carter VA Medical Center, Miami, FL USA; 184 Neurogenetics Program, University of California Los Angeles, Los Angeles, CA USA; 185 Department of Neurology, University of Southern California, Los Angeles, CA USA; 186 Section of Gerontology and Geriatric Medicine Research, Wake Forest School of Medicine, Winston-Salem, NC USA; 187 Department of Medicine, University of Washington, Seattle, WA USA; 188 Department of Neurology, University of California Irvine, Irvine, CA USA; 189 Department of Psychiatry and Behavioral Sciences, Miller School of Medicine, University of Miami, Miami, FL USA; 190 NeuroGenomics and Informatics, Washington University, St. Louis, MO USA; 191 Department of Psychiatry, Washington University in St. Louis, St Louis, MO USA; 192 Department of Psychiatry, University of Texas Southwestern Medical Center, Dallas, TX USA; 193 Alzheimer’s Disease and Memory Disorders Center, Baylor College of Medicine, Houston, TX USA; 194 Department of Population and Data Sciences, University of Texas Southwestern Medical Center, Dallas, TX USA; 195 Center for Translational and Computational Neuroimmunology, Department of Neurology and the Taub Institute for Research in Alzheimer’s Disease and the Aging Brain, Columbia University Irving Medical Center, New York, NY USA; 196 Department of Neurology, University of California Davis, Sacramento, CA USA; 197 Departments of Neurology, Pharmacology and Neuroscience, Texas Tech University Health Science Center, Lubbock, TX USA; 198 Institute for Memory Impairments and Neurological Disorders, University of California Irvine, Irvine, CA USA; 199 Wien Center for Alzheimer’s Disease and Memory Disorders, Mount Sinai Medical Center, Miami Beach, FL USA; 200 Vanderbilt Memory and Alzheimer’s Center, Department of Neurology, Vanderbilt University Medical Center, Nashville, TN USA; 201 Department of Neurology, Mayo Clinic, Jacksonville, FL USA; 202 Rush Institute for Healthy Aging, Department of Internal Medicine, Rush University Medical Center, Chicago, IL USA; 203 Department of Medical and Molecular Genetics, Indiana University, Indianapolis, IN USA; 204 Office of Strategy and Measurement, University of North Texas Health Science Center, Fort Worth, TX USA; 205 Department of Pathology, University of Alabama at Birmingham, Birmingham, AL USA; 206 Department of Neurology, Indiana University, Indianapolis, IN USA; 207 Department of Psychiatry and Hope Center Program on Protein Aggregation and Neurodegeneration, Washington University School of Medicine, St. Louis, MO USA; 208 Department of Neurology, University of Louisville School of Medicine, Louisville, KY USA; 209 C.S. Kubik Laboratory for Neuropathology, Massachusetts General Hospital, Charlestown, MA USA; 210 Department of Neuroscience, Ronald M. Loeb Center for Alzheimer’s Disease, Icahn School of Medicine at Mount Sinai, New York, NY USA; 211 Department of Pathology and Laboratory Medicine, Emory University, Atlanta, GA USA; 212 Emory Alzheimer’s Disease Center, Emory University, Atlanta, GA USA; 213 Department of Pathology and Laboratory Medicine, Indiana University, Indianapolis, IN USA; 214 Department of Radiology, University of Washington, Seattle, WA USA; 215 Department of Neurology, Oregon Health and Science University, Portland, OR USA; 216 Department of Neurology, Portland Veterans Affairs Medical Center, Portland, OR USA; 217 Department of Neurology, Massachusetts General Hospital/Harvard Medical School, Boston, MA USA; 218 Center for Applied Genomics, Children’s Hospital of Philadelphia, Philadelphia, PA USA; 219 Division of Human Genetics, Department of Pediatrics, Perelman School of Medicine, University of Pennsylvania, Philadelphia, PA USA; 220 Department of Pathology (Neuropathology), University of Pittsburgh, Pittsburgh, PA USA; 221 Department of Neurology, Ohio State University, Columbus, OH USA; 222 UCL Institute of Neurology, University College London, London, England UK; 223 Department of Molecular Neuroscience, UCL Institute of Neurology, University College London, London, England UK; 224 Department of Pathology and Laboratory Medicine, University of California Irvine, Irvine, CA USA; 225 Department of Epidemiology and Population Health, Stanford University, Stanford, CA USA; 226 Department of Neurology and Neurological Sciences, Stanford University, Stanford, CA USA; 227 Vanderbilt Genetics Institute, Division of Genetic Medicine, Department of Medicine, Vanderbilt University Medical Center, Nashville, TN USA; 228 Department of Surgery, University of Texas Southwestern Medical Center, Dallas, TX USA; 229 Neurogenomics Division, Translational Genomics Research Institute, Phoenix, AZ USA; 230 Department of Psychiatry, Washington University School of Medicine, St. Louis, MO USA; 231 Hope Center Program on Protein Aggregation and Neurodegeneration, Washington University School of Medicine, St. Louis, MO USA; 232 Department of Genome Sciences, University of Washington, Seattle, WA USA; 233 Department of Medicine (Medical Genetics), University of Washington, Seattle, WA USA; 234 Department of Pathology and Laboratory Medicine, University of California Davis, Sacramento, CA USA; 235 Department of Health Behavior and Health Systems, University of North Texas Health Science Center, Fort Worth, TX USA; 236 Department of Psychiatry, University of Pittsburgh, Pittsburgh, PA USA; 237 Department of Human Genetics, University of Pittsburgh, Pittsburgh, PA USA; 238 Alzheimer’s Disease Research Center, University of Pittsburgh, Pittsburgh, PA USA; 239 Department of Neurology, Albert Einstein College of Medicine, New York, NY USA; 240 Department of Neuroscience, Brigham Young University, Provo, UT USA; 241 Department of Biology, Brigham Young University, Provo, UT USA; 242 Department of Laboratory Medicine and Pathology, University of Washington, Seattle, WA USA; 243 Department of Pathology, Boston University, Boston, MA USA; 244 Department of Neuropsychology, University of California San Francisco, San Francisco, CA USA; 245 Department of Epidemiology, University of Washington, Seattle, WA USA; 246 Department of Neurobiology and Behavior, University of California Irvine, Irvine, CA USA; 247 Department of Neurology, Emory University, Atlanta, GA USA; 248 Kaiser Permanente Washington Health Research Institute, Seattle, WA USA; 249 Department of Pathology, University of Michigan, Ann Arbor, MI USA; 250 National Center for PTSD at Boston VA Healthcare System, Boston, MA USA; 251 Department of Psychiatry, Boston University Chobanian & Avedisian School of Medicine, Boston, MA USA; 252 Department of Psychiatry, Johns Hopkins University, Baltimore, MD USA; 253 Department of Health Management and Policy, School of Public Health, University of North Texas Health Science Center, Fort Worth, TX USA; 254 Department of Neurology, University of Alabama at Birmingham, Birmingham, AL USA; 255 Department of Medicine - Pulmonary, New York University, New York, NY USA; 256 Department of Neurology, Miller School of Medicine, University of Miami, Miami, FL USA; 257 Department of Pathology, University of California San Diego, La Jolla, CA USA; 258 Department of Psychiatry, New York University, New York, NY USA; 259 School of Nursing Northwest Research Group on Aging, University of Washington, Seattle, WA USA; 260 Department of Pathology, Boston University Chobanian & Avedisian School of Medicine, Boston, MA USA; 261 Department of Pathology, Northwestern University Feinberg School of Medicine, Chicago, IL USA; 262 Cognitive Neurology and Alzheimer’s Disease Center, Northwestern University Feinberg School of Medicine, Chicago, IL USA; 263 Weill Institute for Neurosciences, Memory and Aging Center, University of California San Francisco, San Francisco, CA USA; 264 Department of Pathology, University of Southern California, Los Angeles, CA USA; 265 Department of Pathology, Stanford University School of Medicine, Stanford, CA USA; 266 Department of Pathology and Laboratory Medicine and Alzheimer’s Disease Research Center, University of California Irvine, Irvine, CA USA; 267 Department of Neurology, Washington University, St. Louis, MO USA; 268 Department of Cell Biology, Miller School of Medicine, University of Miami, Miami, FL USA; 269 Department of Neurology, University of Texas Southwestern Medical Center, Dallas, TX USA; 270 Department of Research Regulatory Compliance, College of Medicine, Howard University, Washington, DC, USA; 271 Institute for Translational Research, University of North Texas Health Science Center, Fort Worth, TX USA; 272 Center for Mind and Brain and Department of Neurology, University of California Davis, Sacramento, CA USA; 273 Department of Family and Community Medicine, University of Texas Health Science Center San Antonio, San Antonio, TX USA; 274 Department of Laboratory Medicine and Pathology, Mayo Clinic, Rochester, MN USA; 275 Department of Psychiatry and Behavioral Sciences, University of Washington School of Medicine, Seattle, WA USA; 276 Pacific Health Research & Education Institute, Veterans Affairs Pacific Islands Healthcare System, Honolulu, HI USA; 277 Department of Neurology, University of Colorado School of Medicine, Aurora, CO USA; 278 Department of Internal Medicine and Geriatrics, University of North Texas Health Science Center, Fort Worth, TX USA; 279 Department of Medical Education, TCU/UNTHSC School of Medicine, Fort Worth, TX USA; 280 Arizona Alzheimer’s Consortium, Phoenix, AZ USA; 281 Banner Alzheimer’s Institute, Phoenix, AZ USA; 282 Department of Psychiatry, University of Arizona, Phoenix, AZ USA; 283 Alzheimer’s Disease Center, New York University, New York, NY USA; 284 Department of Biostatistics, O’Donnell School of Public Health, the University of Texas Southwestern Medical Center, Dallas, TX USA; 285 Department of Neurology, University of California Los Angeles, Los Angeles, CA USA; 286 Tanz Centre for Research in Neurodegenerative Disease, University of Toronto, Toronto, Ontario, Canada; 287 Departments of Psychiatry, Medicine, Family and Community Medicine, and the Glenn Biggs Institute for Alzheimer’s and Neurodegenerative Diseases, UT Health Science Center at San Antonio, San Antonio, TX USA; 288 Department of Neurology, Barrow Neurological Institute St. Joseph’s Hospital and Medical Center, Phoenix, AZ USA; 289 Department of Medical Genetics, University of British Columbia, Vancouver, British Columbia Canada; 290 Department of Radiology and Imaging Sciences, Indiana University, Indianapolis, IN USA; 291 Department of Pathology (Neuropathology), Rush University Medical Center, Chicago, IL USA; 292 Department of Psychiatry, University of Southern California, Los Angeles, CA USA; 293 Cambridge Institute for Medical Research, University of Cambridge, Cambridge, England UK; 294 Faculty of Medicine, Department of Medicine (Neurology), University of Toronto, Toronto, Ontario, Canada; 295 Maya Angelou Center for Health Equity, Wake Forest University School of Medicine, Winston-Salem, NC, USA; Center for Outreach in Alzheimer’s, Aging and Community Health at North Carolina A&T State University, Greensboro, NC USA; 296 Center for Applied Health Research, Baylor Scott & White Health, Temple, TX USA; 297 Center for Population Health and Aging, Texas A&M University Health Science Center, Lubbock, TX USA; 298 College of Medicine, Texas A&M University Health Science Center, College Station, TX USA; 299 Program in Cellular Neuroscience, Neurodegeneration and Repair, Yale University School of Medicine, New Haven, CT USA; 300 Department of Psychiatry and Human Behavior, and Institute for Memory Impairments and Neurological Disorders (UCI-MIND), University of California Irvine, Irvine, CA USA; 301 Renaissance Computing Institute, University of North Carolina Chapel Hill, Chapel Hill, NC USA; 302 Department of Pathology, Johns Hopkins University, Baltimore, MD USA; 303 Department of Neuroscience, College of Medicine, University of Kentucky, Lexington, KY USA; 304 Department of Pathology and Laboratory Medicine, University of California Los Angeles, Los Angeles, CA USA; 305 Department of Psychiatry and Behavioral Sciences, Northwestern University Feinberg School of Medicine, Chicago, IL USA; 306 Department of Psychiatry and Behavioral Sciences, Duke University, Durham, NC USA; 307 Department of Medicine, Duke University, Durham, NC USA; 308 Department of Biostatistics, University of Washington, Seattle, WA USA; 309 Department of Genetics, University of North Carolina Chapel Hill, Chapel Hill, NC USA; 310 Department of Neurology, Section of Gerontology and Geriatric Medicine Research, Wake Forest School of Medicine, Winston-Salem, NC USA; 311 Department of Psychiatry, New York University Grossman School of Medicine, New York, NY USA; 312 Center for Cognitive Neurology and Departments of Neurology and Pathology, New York University Grossman School of Medicine, New York, NY USA; 313 Department of Pathology, Oregon Health and Science University, Portland, OR USA; 314 National Institute of Neurological Disorders and Stroke, National Institutes of Health, Bethesda, MD USA; 315 Department of Pathology, Case Western Reserve University, Cleveland, OH USA; 316 Department of Ophthalmology, Boston University Chobanian & Avedisian School of Medicine, Boston, MA USA; 317 Department of Epidemiology, Columbia University, New York, NY USA; 318 Social Sciences & Health Policy, Wake Forest School of Medicine, Winston-Salem, NC USA; 319 Department of Biostatistics, University of Kentucky, Lexington, KY USA; 320 Department of Epidemiology, Boston University School of Public Health, Boston, MA USA; 321 The Glenn Biggs Institute for Alzheimer’s and Neurodegenerative Diseases, The University of Texas Health Science Center at San Antonio, San Antonio, TX USA; 322 Department of Biochemistry and Structural Biology, The University of Texas Health Science Center at San Antonio, San Antonio, TX USA; 323 Department of Population Health Sciences, University of Texas Health Science Center, San Antonio, TX USA; 324 Department of Epidemiology, Erasmus MC, University Medical Center, Rotterdam, The Netherlands; 325 University of Bordeaux, Inserm, Bordeaux Population Health Research Center, UMR 1219, Bordeaux, France; 326 Cardiovascular Health Research Unit, Department of Medicine, University of Washington, Seattle, WA USA; 327 Framingham Heart Study, Framingham, MA USA; 328 Department of Neurology, Boston University School of Medicine, Boston, MA USA; 329 Faculty of Medicine, University of Iceland, Reykjavik, Iceland; 330 Icelandic Heart Association, Kopavogur, Iceland; 331 Laboratory of Epidemiology and Population Sciences, Intramural Research Program, National Institute of Aging, National Institutes of Health, Bethesda, MD USA; 332 Human Genetics Center, School of Public Health, The University of Texas Health Science Center at Houston, Houston, TX USA; 333 Department of Psychiatry and Psychotherapy, University Medicine Greifswald, Greifswald, Germany; 334 German Center for Neurodegenerative Diseases (DZNE), Site Rostock/ Greifswald, Rostock, Germany; 335 Department of Health Systems and Population Health, University of Washington, Seattle, WA USA; 336 Human Genome Sequencing Center, Baylor College of Medicine, Houston, TX USA; 337 Department of Epidemiology, Human Genetics and Environmental Sciences, University of Texas Health Science Center at Houston, School of Public Health, Houston, TX USA; 338 Memory Impairment and Neurodegenerative Dementia (MIND) Center and Department of Medicine, University of Mississippi Medical Center, Jackson, MS USA; 339 Department of Neurology, School of Medicine, University of Pittsburgh, Pittsburgh, PA USA; 340 Big Data Institute, Li Ka Shing Centre for Health Information and Discovery, Old Road Campus, Headington, Oxford, UK; 341 Department of Neurology, Erasmus University Medical Centre, Rotterdam, The Netherlands; 342 Institute of Molecular Medicine, McGovern Medical School, The University of Texas Health Science Center at Houston, Houston, TX USA; 343 Department of Neurology, UT Health San Antonio, San Antonio, TX USA; 344 Human Genetics, School of Life Sciences, University of Nottingham, Nottingham, UK; 345 Nottingham Trent University, Nottingham, UK; 346 Human Genetics, University of Nottingham, Nottingham, UK; 347 Clinical and Experimental Science, Faculty of Medicine, University of Southampton, Southampton, UK; 348 School of Health Sciences, Bangor University, Bangor, UK; 349 Wales Centre for Ageing & Dementia Research, Bangor, UK; 350 Faculty of Medical & Health Sciences, University of Auckland, Auckland, New Zealand; 351 Faculty of Medicine, Health and Life Science, Swansea University, Swansea, UK; 352 Institute of Public Health, University of Cambridge, Cambridge, UK; 353 MRC Prion Unit at UCL, UCL Institute of Prion Diseases, London, UK; 354 Dementia Research Centre, UCL, London, UK; 355 CAEBI—Centro Andaluz de Estudios Bioinformáticos, Sevilla, Spain; 356 Grupo de Medicina Xenómica, Fundación Pública Galega de Medicina Xenómica, Santiago de Compostela, Spain; 357 Grupo de Medicina Xenómica, Centro de Investigación Biomédica en Red de Enfermedades Raras (CIBERER), Universidade de Santiago de Compostela (CIMUS), Santiago de Compostela, Spain; 358 Fundación Pública Galega de Medicina Xenómica- Instituto de Investigación Sanitaria de Santiago (IDIS), Santiago de Compostela, Spain; 359 Instituto de Biomedicina de Sevilla (IBIS), Universidad de Sevilla, Hospital Universitario Virgen de Valme, CIBERINFEC, Sevilla, Spain; 360 Depatamento de Bioquímica Médica, Biología Molecular e Inmunología, Facultad de Medicina, Universidad de Sevilla, Sevilla, Spain; 361 Centro de Investigación Biomédica en Red de Enfermedades Infecciosas (CIBERINFEC), Instituto de Salud Carlos III, Madrid, Spain; 362 Alzheimer Research Center & Memory Clinic, Instituto Andaluz de Neurociencia, Málaga, Spain; 363 Hospital Universitario Ramon y Cajal, IRYCIS, Madrid, Spain; 364 Unidad de Demencias, Servicio de Neurología y Neurofisiología, Instituto de Biomedicina de Sevilla (IBiS), Hospital Universitario Virgen del Rocío/CSIC/Universidad de Sevilla, Seville, Spain; 365 Genomcore, Esplugues de Llobregat, Barcelona, Spain; 366 Department of Neurology, II B Sant Pau, Hospital de la Santa Creu i Sant Pau, Universitat Autònoma de Barcelona, Barcelona, Spain; 367 Department of Neurology, La Paz University Hospital, Instituto de Investigación Sanitaria del Hospital Universitario La Paz, IdiPAZ, Madrid, Spain; 368 Hospital La Paz Institute for Health Research, IdiPAZ, Madrid, Spain; 369 Department of Neurology, La Paz University Hospital, Madrid, Spain; 370 Servicio de Neurología. Hospital Universitario Central de Asturias, Oviedo, Spain; 371 Departamento de Medicina, Universidad de Oviedo, Oviedo, Spain; 372 Department of Neurosciences, Faculty of Medicine and Nursery, University of the Basque Country, San Sebastián, Spain; 373 CIEN Foundation/Queen Sofia Foundation Alzheimer Center/Instituto de Salud Carlos III, Madrid, Spain; 374 UFIEC, Instituto de Salud Carlos III, Madrid, Spain; 375 Department of Immunology, Hospital Universitario Doctor Negrín, Las Palmas de Gran Canaria, Las Palmas, Spain; 376 Department of Complex Trait Genetics, Center for Neurogenomics and Cognitive Research, Amsterdam Neuroscience, Vrije Universiteit Amsterdam, Amsterdam, The Netherlands; 377 Department of Child and Adolescent Psychiatry and Pediatric Psychology, Section Complex Trait Genetics, Amsterdam Neuroscience, Vrije Universiteit Medical Center, Amsterdam University Medical Center, Amsterdam, The Netherlands; 378 Amgen deCODE genetics, Sturlugata 8, Reykjavík, Iceland; 379 Department of Geriatric Medicine, Landspitali University Hospital, Reykjavik, Iceland; 380 Department of Medical Epidemiology and Biostatistics, Karolinska Institutet, Stockholm, Sweden; 381 University of Colorado Boulder, Institute for Behavior Genetics and Department of Psychology and Neuroscience, Boulder, CO USA; 382 Neuropsychiatric Epidemiology Unit, Department of Psychiatry and Neurochemistry, Institute of Neuroscience and Physiology, Sahlgrenska Academy, Centre for Ageing and Health (AgeCap) at the University of Gothenburg, Mölndal, Sweden; 383 Region Västra Götaland, Sahlgrenska University Hospital, Neuropsychiatry Clinic, Gothenburg, Sweden; 384 Region Västra Götaland, Sahlgrenska University Hospital, Psychiatry, Psychosis Clinic, Gothenburg, Sweden; 385 Clinical Neurochemistry Laboratory, Sahlgrenska University Hospital, Mölndal, Sweden; 386 Department of Psychiatry and Neurochemistry, Institute of Neuroscience and Physiology, the Sahlgrenska Academy at the University of Gothenburg, Mölndal, Sweden; 387 Paris Brain Institute, ICM, Pitié-Salpêtrière Hospital, Sorbonne University, Paris, France; 388 Neurodegenerative Disorder Research Center, Division of Life Sciences and Medicine, and Department of Neurology, Institute on Aging and Brain Disorders, University of Science and Technology of China and First Affiliated Hospital of USTC, Hefei, P.R. China; 389 Department of Neurodegenerative Disease, UCL Institute of Neurology, Queen Square, London, UK; 390 UK Dementia Research Institute at UCL, London, UK; 391 Hong Kong Center for Neurodegenerative Diseases, Clear Water Bay, Hong Kong China; 392 Wisconsin Alzheimer’s Disease Research Center, University of Wisconsin School of Medicine and Public Health, University of Wisconsin-Madison, Madison, WI USA; 393 HUNT Center for Molecular and Clinical Epidemiology, Department of Public Health and Nursing, NTNU, Norwegian University of Science and Technology, Trondheim, Norway; 394 HUNT Research Centre, Department of Public Health and Nursing, NTNU, Norwegian University of Science and Technology, Levanger, Norway; 395 Department of Research, St. Olavs Hospital, Trondheim University Hospital, Trondheim, Norway; 396 Department of Research and Innovation, Division of Clinical Neuroscience, Oslo University Hospital, Oslo, Norway; 397 Department of Neurology, Oslo University Hospital, Oslo, Norway; 398 Clinic of Medicine, St. Olavs Hospital, Trondheim University Hospital, Trondheim, Norway; 399 Norwegian National Centre for Ageing and Health, Vestfold Hospital Trust, Tønsberg, Norway; 400 Institute for Clinical Medicine, University of Oslo, Oslo, Norway; 401 Department of Geriatric Medicine, Oslo University Hospital, Oslo, Norway; 402 Department of Neurology, Akershus University Hospital, Lørenskog, Norway; 403 Centre of Age-Related Medicine, Stavanger University Hospital, Stavanger, Norway; 404 Institute of Psychiatry, Psychology and Neurosciences, King’s College London, London, UK; 405 Department of Medical Genetics, Oslo University Hospital, Oslo, Norway; 406 Centre for Precision Psychiatry, Division of Mental Health and Addiction, University of Oslo, and Oslo University Hospital, Oslo, Norway; 407 Department of Research and Innovation, Helse Fonna, Haugesund, Norway; 408 Department of Clinical Medicine 1 (K1), University of Bergen, Bergen, Norway; 409 Department of Neuromedicine and Movement Science (INB), NTNU, Faculty of Medicine and Health Sciences, Trondheim, Norway; 410 Department of Geriatric Medicine, Clinic of Medicine, St. Olavs Hospital, Trondheim University Hospital, Trondheim, Norway; 411 Department of Neurology, St Olav’s Hospital, Trondheim University Hospital, Trondheim, Norway; 412 Department of Neuromedicine and Movement Science, Faculty of Medicine and Health Sciences, Norwegian University of Science and Technology (NTNU), Trondheim, Norway

**Keywords:** Alzheimer's disease, Genome-wide association studies

## Abstract

To better characterize the genetic architecture underlying Alzheimer’s disease (AD) and related dementias (ADRD), we performed a meta-analysis of European-ancestry genome-wide association studies in 128,681 cases or proxy cases of ADRD and 849,833 (proxy) controls. We identified 91 genetic loci associated with ADRD risk, of which 16 are new and 56 are specifically detected in clinically diagnosed AD cases. We also provide a list of 18 loci (15 new) requiring further external validation. A polygenic score combining the effects of ADRD loci other than *APOE* was primarily associated with AD rather than non-AD pathology. Individuals in the tenth decile of the score exhibited a twofold increased risk of presenting with Braak neurofibrillary tangles stage of >4 and moderate-to-severe neuritic amyloid plaque pathology at death compared to individuals in the median score group. In conclusion, our study validated a large number of loci associated with the risk of clinically diagnosed AD, while further investigations are required to confirm the impact of the other loci on AD clinical diagnosis and of each locus on AD pathology.

## Abstract (web summary)

An updated meta-analysis of genome-wide association studies identified 91 risk loci for Alzheimer’s disease (AD) and related dementias among individuals of European ancestry, of which 56 loci were specifically detected in clinically diagnosed AD cases.

## Main

More than 90 genetic loci are associated with Alzheimer’s disease and related dementia (ADRD). Most of them were identified through large-scale genome-wide association studies (GWASs) performed in European-ancestry samples by, among others, the International Genomics of Alzheimer’s Project (IGAP), the European Alzheimer and Dementia Biobank (EADB) and the Psychiatric Genomics Consortium (PGC)—AD Working Group (PGC-ALZ)^1–7^. Although the latest GWASs on ADRD partly overlapped, they used different imputation panels or analytical approaches and some results were discordant across studies. They all included, to varying extents, large biobank samples using International Classification of Diseases codes to identify AD cases, proxy ADRD cases (that is, individuals reporting at least one parent or sibling with dementia) or both. This strategy increases the power to identify AD loci but also can blur the distinction between AD and non-AD dementia signals, as the AD phenotype definition is less specific in those samples. To better characterize the genetic architecture and pathophysiology underlying AD and ADRD, we joined efforts across the three consortia to perform a consensus meta-analysis of ADRD GWAS across all our samples of European ancestry, the UK Biobank (UKBB) and FinnGen. To further delineate the impact of known genomic loci on AD compared to ADRD, we performed sensitivity analyses by excluding proxy or large biobank samples.

**Fig. 1:**
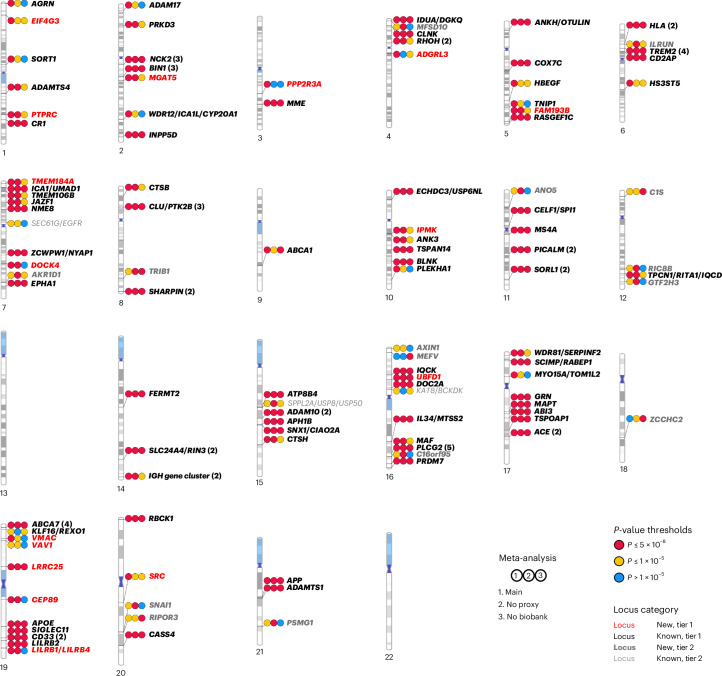
Ideogram of the tier 1 and 2 loci. Tier 1 loci are considered genuine GWS signals, with *P* ≤ 5 × 10^−8^ in the unconditional analysis and *P* ≤ 1 × 10^−7^ in conditional analyses within or across loci. Tier 2 loci are GWS signals requiring further external validation because (1) the *P* value was > 1 × 10^−7^ after conditional analyses or (2) they were GWS only in the sensitivity no-proxy and no-biobank meta-analyses. For each locus, the figure shows the *P*-value categories for the association with ADRD or AD risk in the main, no-proxy and no-biobank meta-analyses—*P* ≤ 5 × 10^−8^, *P* ≤ 1 × 10^−5^, *P* > 1 × 10^−5^ (two-sided raw *P* values derived from a fixed-effect meta-analysis with an inverse-variance-weighted approach). We considered the *P* value of the lead variant when the locus was detected in the meta-analysis and otherwise the minimum *P* value across all index variants of the locus. New tier 1 loci are in bold red, known tier 1 loci in bold black, known tier 2 loci in gray and new tier 2 loci in bold gray. Numbers in parentheses refer to the number of GWS independent signals within the locus according to the main meta-analysis.

The meta-analysis included 72,721 AD cases, 55,960 proxy ADRD cases, 614,267 controls and 235,566 proxy controls from 52 studies, corresponding to a rough effective sample size of 230,631 (Supplementary Note and Supplementary Table 1). After quality control, we considered associations for 20,045,120 variants (Supplementary Figs. 1 and 2). We identified 91 genome-wide significant (GWS) loci (*P* ≤ 5 × 10^−8^, defined as ‘tier 1’), of which 16 loci (*EIF4G3*, *PTPRC*, *MGAT5*, *PPP2R3A*, *ADGRL3*, *FAM193B*, *TMEM184A*, *DOCK4*, *IPMK*, *UBFD1*, *VMAC*, *VAV1*, *LRRC25*, *CEP89*, *LILRB1*/*LILRB4* and *SRC*) were new in European-ancestry samples at the time of analysis, although *ADGRL3* was recently identified in a multi-ancestry GWAS^8^ (Fig. 1, Supplementary Tables 2 and 3 and Supplementary Figs. 3–55). After the stepwise conditional analysis, we identified 25 independent secondary signals across 16 loci (Fig. 1, Supplementary Table 3 and Supplementary Figs. 3–55). Compared to refs. ^4–6,9^, the independent association signals detected in the *CD33*, *HLA*, *PICALM* and *RHOH* loci are new and we detected additional independent signals in *ABCA7*, *BIN1*, *PTK2B*/*CLU*, *NCK2* and *PLCG2* (Fig. 1). While some main and secondary signals are likely linked to the same gene—for example, the low-frequency variants in *SORL1* and *ABCA7*—some may be linked to two different genes in the same locus. Of note, in addition to the 91 tier 1 loci, we detected five loci in which significance decreased after conditional analyses (*P* > 1 × 10^−7^) performed either within or across loci, suggesting a slight inflation of the unconditional analysis (Fig. 1 and Supplementary Tables 2–4). Those loci require external validation and were thus classified as ‘tier 2’. Three were known (*SEC61G/EGFR*, *SPPL2A*/*USP8*/*USP50*, *KAT8*/*BCKDK*) and two were new in European-ancestry samples (*TRIB1* and *AXIN1*), although *TRIB1* was identified in a recent multi-ancestry GWAS^8^. Additionally, four loci identified in the two previous largest ADRD GWAS meta-analyses on European-ancestry samples^5,6^ (*HAVCR2*, *SLC2A4RG*/*LIME1*, *FOXF1* and *NTN5)* were not GWS in the EADB–IGAP–PGC meta-analysis (Supplementary Table 5 and Supplementary Fig. 56).

We assessed the robustness of the signals according to the AD diagnosis quality by excluding proxy ADRD samples or large biobank samples from the meta-analysis. Overall, the genetic correlation among the three AD phenotypes thus defined was above 0.97 (Supplementary Table 6). Among the 91 tier 1 loci, 75 (82.4%) were GWS in the no-proxy meta-analysis, and 56 (61.5%) in the no-biobank meta-analysis focusing on clinically diagnosed AD cases (Fig. 1 and Supplementary Tables 2 and 7). Ten of the 75 loci detected in both the main and no-proxy meta-analyses were new—*PTPRC*, *MGAT5*, *FAM193B*, *TMEM184A*, *DOCK4*, *IPMK*, *UBFD1*, *LRRC25*, *CEP89* and *LILRB1/LILRB4*. The *UBFD1* and *LRRC25* new loci were also GWS in the no-biobank meta-analysis, while 12 known ADRD loci are now GWS after focusing on clinically diagnosed AD cases compared with refs. ^3,4,6^ (*NCK2*, *MME*, *ANKH/OTULIN*, *ABCA1*, *BLNK*, *IGH*, *ATP8B4*, *SNX1/CIAO2A*, *IL34*/*MTSS2*, *SIGLEC11*, *RBCK1* and *APP*). We further examined putative signals detected exclusively in the sensitivity meta-analyses—nine loci were identified only in the no-proxy meta-analysis, while four loci were identified only in the no-biobank meta-analysis (Fig. 1, Supplementary Tables 2 and 7 and Supplementary Figs. 57–61). All 13 of these loci were new and classified as ‘tier 2’. The Supplementary Note further describes the GWAS results and provides putative secondary signals at a more lenient significance threshold of *P* < 1 × 10^−5^ (Supplementary Table 3).

Among the index variants of the main or secondary tier 1 signals, three were missense variants with a REVEL score of >0.25 (in the *MME*, *TREM2* and *ABCA7* loci); only seven index variants were rare, with minor allele frequency (MAF) of <1%, in the *SORT1*, *NCK2*, *ADGRL3*, *TREM2*, *PLCG2* and *ABCA7* loci (Supplementary Tables 2 and 3). We further assessed the impact of rare variants within the new tier 1 loci on AD risk using a previous gene-based analysis of our samples’ sequencing data. In the *SRC* gene, loss-of-function variants and missense variants with a REVEL score of >50 were jointly associated with AD risk (odds ratio (OR) = 4.23, 95% confidence interval (CI) = 2.04–8.79, *P* = 1.06 × 10^−4^; Supplementary Table 8) in the Alzheimer Disease European Sequencing (ADES) - Alzheimer’s Disease Sequencing Project (ADSP) summary statistics^10^.

As in previous GWAS findings, genes enriched for ADRD or AD association signals in the main, no-proxy and no-biobank meta-analyses were overexpressed in microglia across four datasets spanning different brain regions (Supplementary Note, Supplementary Tables 9 and 10 and Supplementary Figs. 62–64). Similar to previous GWAS results, association signals were significantly enriched in biological pathways related to tau, amyloid, lipids, immunity or endosome/lysosome, in the main, no-proxy and no-biobank meta-analyses (Supplementary Table 11). By performing a phenome-wide association study, we also linked some of the new loci (both tiers) to pathways related to tau, lipids or immunity (Supplementary Tables 12–14). For example, the *C16orf95* locus has been associated with phosphorylated tau levels in cerebrospinal fluid as well as with ventricular volume (Supplementary Note)^11,12^. This latter observation supports the validity of some of the tier 2 loci. After exclusion of *APOE*, the no-proxy ADRD phenotype was significantly (*P* ≤ 3.13 × 10^−3^) genetically correlated with Lewy body dementia (*r* = 0.64, 95% CI = 0.37–0.92), amyotrophic lateral sclerosis (*r* = 0.29, 95% CI = 0.18–0.40), Parkinson’s disease (*r* = 0.16, 95% CI = 0.06–0.27) and educational attainment (*r* = −0.12, 95% CI = −0.17 to –0.07; Supplementary Table 6). These results, consistent with previous studies^13,14^, were similar when considering the main and no-biobank summary statistics except for educational attainment when using the main meta-analysis results (*r* = −0.02, 95% CI = −0.07 to 0.02). This is in accordance with previous reports of biases observed with genome-wide summary statistics of studies including proxy cases^14–16^.

**Fig. 2:**
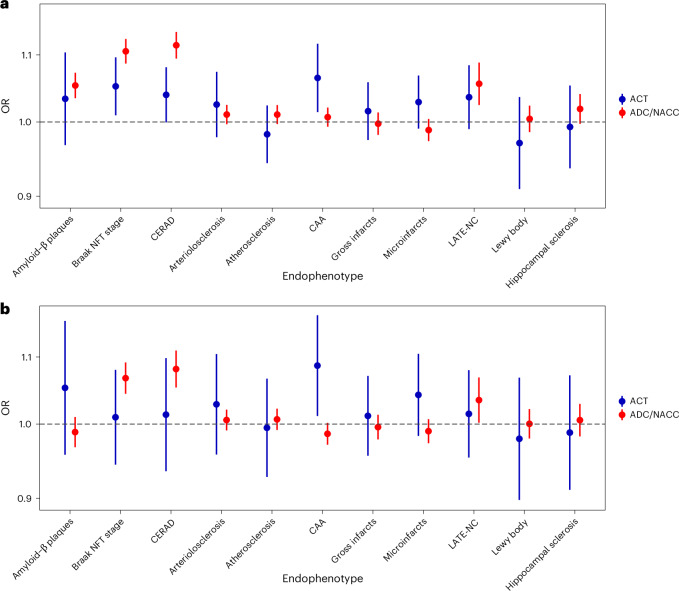
Association of the main ADRD PGS with 11 neuropathology endophenotypes in the ACT and ADC/NACC datasets. **a**,**b**, Analyses were performed with minimal adjustment on age at death, sex, number of *APOE* ε4 and ε2 alleles, PCs and centers (**a**) and additional adjustment on AD neuropathology endophenotypes (**b**). Dots represent OR and bars indicate 95% CI. NFT, neurofibrillary tangles; CAA, cerebral amyloid angiopathy.

**Fig. 3:**
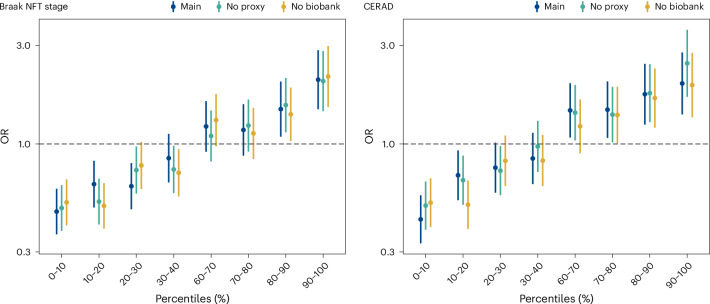
Association of the main, no-proxy and no-biobank ADRD PGS deciles with Braak stage and CERAD score in the ADC/NACC dataset. Braak stage was dichotomized into stages 0–3 (*n* = 1,113) versus stages 4–6 (*n* = 4,680) groups. CERAD stage was dichotomized into none/mild (*n* = 1,062) versus moderate/severe (*n* = 4,738) groups. Analyses were adjusted for age at death, sex, number of *APOE* ε4 and ε2 alleles, PCs and centers. The reference is the median (40–60%) quintile. Dots represent OR and bars indicate 95% CI.

Genetic correlation with ADRD could not be reliably assessed for most of the 11 neuropathology endophenotypes (NPEs) examined, due to limited study sample sizes and/or low heritability of these traits (Supplementary Table 6). These NPEs comprised the following: (1) three AD-related NPEs—Braak neurofibrillary tangles stage, amyloid-β plaques and the CERAD score for neuritic amyloid plaques; (2) five cerebrovascular NPEs—arteriolosclerosis, circle of Willis atherosclerosis, cerebral amyloid angiopathy, gross infarcts and microinfarcts, and (3) three non-AD NPEs—limbic-predominant age-related TDP-43 encephalopathy neuropathological change (LATE-NC), Lewy bodies and hippocampal sclerosis. To better study the genetic link between ADRD and NPEs, we constructed three polygenic scores (PGSs) based on the tier 1 main and secondary signals (excluding *APOE*) detected in the main, no-proxy and no-biobank meta-analyses, respectively (Supplementary Table 15). We then assessed the association of these PGSs with the 11 NPEs in the Adult Changes in Thought (ACT) cohort (*n* = 677, including 12.9% with dementia) and in the Alzheimer’s Disease Centers/National Alzheimer’s Coordinating Center (ADC/NACC) dataset (*n* = 5,808, including 82.7% with dementia; Supplementary Table 16)^17^. The main score was significantly associated (*P* ≤ 2.27 × 10^−3^) with the three AD-related NPEs and with LATE-NC in the ADC/NACC dataset, and the signals were in the same direction in the smaller ACT study (Fig. 2a, Supplementary Table 17 and Supplementary Supplementary Note). None of the scores was significantly associated with cerebrovascular NPEs, hippocampal sclerosis or Lewy bodies (Fig. 2a and Supplementary Table 17), while the genetic correlation between ADRD and Lewy body dementia was significant after the exclusion of *APOE*. This may indicate common pathological pathways underlying conversion from the pathology to AD or Lewy body dementia. However, we cannot exclude other explanations, such as a lack of statistical power of the PGS analysis or misdiagnoses among AD and Lewy body dementia cases, impacting genetic correlation. Results were similar across the main, no-proxy and no-biobank PGS (Supplementary Table 17). After adjustment for the AD-related NPEs, only the associations with Braak stage and CERAD score remained significant in ADC/NACC (Fig. 2b and Supplementary Table 17). The association with LATE-NC remained significant at the nominal level only (*P* = 3.78 × 10^−2^; Supplementary Table 17); a larger sample size is required to assess whether the association with LATE-NC is due to or not to the frequent co-occurrence of LATE-NC and AD NPEs (Supplementary Note). There was no significant interaction (*P* ≤ 2.27 × 10^−3^) between the PGS and the number of *APOE* ε4 and ε2 alleles for Braak stage and CERAD score (Supplementary Table 17). In the ADC/NACC dataset, compared to the individuals with a main PGS in the median quintile (40–60%), individuals in the tenth decile had a risk increased by 2.05-fold (95% CI = 1.47–2.85) and 1.96-fold (95% CI = 1.39–2.78) for Braak stage of >4 and moderate-to-severe neuritic amyloid plaque pathology at death, respectively, while individuals in the first decile had a risk decreased by 0.47-fold (95% CI = 0.37–0.61) and 0.43-fold (95% CI = 0.33–0.56), respectively (Fig. 3, Supplementary Tables 18 and 19 and Supplementary Note). Compared with a model considering only age at death, sex and the number of *APOE* ε4 and ε2 alleles, the main PGS allowed a significant improvement (*P* ≤ 2.27 × 10^−3^) in discrimination measured by the area under the receiver operating characteristic curve (AUC) for both Braak stage and CERAD score in the ADC/NACC dataset (Supplementary Table 20). However, the variance explained by the score remained low—Nagelkerke’s pseudo-*R*-squared (*R*^2^) was 3.98% and 4.37% for Braak stage and CERAD score, respectively, while the variance explained on the liability scale varied between 2.43% and 3.6% for Braak stage, and between 3.32% and 4.93% for CERAD score, depending on the population prevalence considered. The discriminative power, as measured by AUC, was similar for the main, no-proxy and no-biobank PGS (*P* > 0.05; Supplementary Table 21).

In summary, this consensus meta-analysis identified 91 genetic loci associated with ADRD risk, including 16 new loci in European-ancestry samples, and 56 of the loci were associated with the risk of clinically diagnosed AD. We also further characterized the impact of known loci by validating—or not—their association with ADRD and AD risk in larger samples and by identifying new secondary signals in some of them. Except for genetic correlation with education, our results were consistent across the main, no-proxy and no-biobank meta-analyses, and the three PGSs, excluding *APOE*, were primarily associated with AD rather than non-AD pathology. However, assessing the sensitivity of GWAS or GWAS secondary analyses (especially those based on genome-wide statistics^14^) in clinically diagnosed cases will become increasingly important as the proportion of proxy and biobank cases included in the GWAS increases. This will be made easier by the release of the no-proxy and no-biobank summary statistics. Additionally, several ADRD loci were significantly associated with non-AD NPEs, but not with AD NPEs (Supplementary Table 14)^17^. Such results are difficult to interpret, considering the limited statistical power of NPE GWASs to detect variants with small effects on ADRD risk. Further studies using larger numbers of well-characterized AD patients and neuropathological data will thus be required to more precisely delineate the impact of each of the loci on AD pathology versus other neuropathologies. Follow-up analyses of rare damaging and structural variants, using sequencing data and functional studies, will provide further insights into the biological impact of these loci on ADRD and AD.

## Methods

### Samples

We analyzed genotyping data from European-ancestry samples across 52 studies—46 case–control or cohort studies, 2 family studies (NIA-LOAD and Framingham Heart Study (FHS)) and 4 large biobanks (UKBB, FinnGen, deCODE and HUNT). The samples are described in Supplementary Table 1 and in the Supplementary Note. In the UKBB, proxy ADRD cases included participants who reported at least one biological relative (parent or siblings) affected with dementia, either at baseline or follow-up (Supplementary Note). Eleven NPEs were measured in autopsied individuals from the ACT and ADC/NACC studies (Supplementary Table 16 and Supplementary Note). The NPE definitions and harmonization approach are discussed in ref. ^17^. Written informed consent was obtained from all study participants or, for those with substantial cognitive impairment, from a caregiver, legal guardian or other proxy. The appropriate review boards from the ADGC, Bonn, CHARGE, EADB, EADI, GERAD, GR@ACE/DEGESCO and PGC-ALZ reviewed and approved the study protocol. Researchers from each participating consortium were actively involved throughout the research process.

### Quality control and imputation

Classical quality control protocols were applied to samples and autosomal variants in each study (Supplementary Note). Most of the samples were imputed with the TOPMed reference panel^18,19^; one study was imputed with the Haplotype Reference Consortium panel^20^, while the UKBB, FinnGen and deCODE biobanks were imputed using study-specific reference panels (Supplementary Table 22).

### GWAS and meta-analyses

Associations between each autosomal variant and ADRD risk were tested within each study under an additive genetic model. Logistic regression was used in most studies. When necessary, relatedness was accounted for using generalized estimating equations or logistic mixed models (Supplementary Note). Analyses were adjusted for principal components (PCs) and center/batches. In a few studies, adjustment was also performed for sex, age or both (Supplementary Table 22). In deCODE, correction for inflation of test statistics due to relatedness and population stratification was performed using the intercept estimate (1.30) from linkage disequilibrium (LD) score regression^21^. In the UKBB-proxy analysis, effect sizes and standard errors were corrected by a factor of two^22,23^. Across all studies, we filtered out duplicated variants and those with (1) missing data for effect size, s.e. or *P* value; (2) an absolute effect size >5 and (3) imputation quality <0.3 (0.8 for the GenADA study). For deCODE and UKBB, data were analyzed in the GRCh37 assembly, and we excluded variants for which conversion of position or alleles from GRCh37 to GRCh38 was not possible or was ambiguous^6^. For the UKBB and the EADB-core HRC study, variants with very large differences in frequency between the TOPMed reference panel and the reference panels used to perform imputation were also excluded^6^.

### Meta-analyses

Results were combined across studies using a fixed-effects meta-analysis with an inverse-variance weighted approach, as implemented in the METAL (v2020-05-05) software^24^. In the main meta-analysis, all studies were included, and within each study we filtered out variants with an effective allele count (defined as the product of the imputation quality and the expected minimum minor allele count between cases and controls) of <5 (ref. ^25^). After meta-analysis, we filtered out (1) variants with frequency amplitude of >0.4 (defined as the difference between the maximum and minimum frequencies across all the studies) and (2) variants analyzed in <40% effective number of cases. In each study, the effective number of cases was defined as the raw number of cases, except in the UKBB proxy, where it was computed by dividing the raw number of proxy cases by four^22,26^. Several sensitivity meta-analyses were conducted as follows by

1. excluding the UKBB-proxy study (no-proxy meta-analysis). The UKBB study was included in the meta-analysis, but only diagnosed cases were considered (Supplementary Note and Supplementary Table 1). Variants with positions of >80 Mb on chromosome 11 were inadvertently missing from the UKBB-diagnosed summary statistics, resulting in a minor loss of power (~6% effective sample size) in a small genomic region (~2%) for the no-proxy sensitivity analyses; this had minimal impact on the results (Supplementary Note). Corrected summary statistics are provided in the GWAS Catalog and on NIAGADS (Data availability);
2. excluding the UKBB, FinnGen, deCODE and HUNT studies, corresponding to large biobanks using International Classification of Diseases codes to identify AD cases (no-biobank meta-analysis; Supplementary Table 1); and
3. removing the per-study variant filtering on effective allele count, except in the FHS study (Supplementary Note and Supplementary Table 23).

Additionally, we assessed the sensitivity of results for GWS lead variants from the main meta-analysis after adjustment for age and sex, and after adjustment for age, sex and the number of *APOE* ε4 and ε2 alleles (Supplementary Table 24).

### Loci definition

We selected variants with GWS signals and also suggestive variants (*P* ≤ 1 × 10^−5^) located within ±500 kb of a GWS variant and analyzed in ≥70% effective number of cases. LD across the variants was computed in the EADB-core dataset using genotype dosages with LDstore 2 (ref. ^27^). For variants not available in EADB-core, LD was computed in 1000 Genomes European samples (v3) using emeraLD^28^, which took phase into account. Variants not available in EADB-core or 1000 Genomes were considered as having no LD with other variants. Loci and their boundaries were then defined based on LD and distance between GWS and suggestive variants using a clumping approach similar to that described in ref. ^29^ (Supplementary Note).

In each locus, the variant with the lowest *P* value (or the highest absolute effect size in case of equal *P* values) was defined as the lead variant. None of the variants with missing LD in both EADB-core and 1000 Genomes was selected as a lead variant.

A locus was considered known if a variant previously associated with AD at the GWS level, according to the GWAS Catalog (version e112_r2024-07-08), was located in this locus^30^. For that, we restricted the GWAS Catalog to ‘MAPPED_TRAIT’ equal to ‘late-onset Alzheimers disease’, ‘Alzheimer disease’, ‘Alzheimer disease, family history of Alzheimer’s disease’, ‘family history of Alzheimer’s disease’ or ‘Alzheimer disease, dementia, family history of Alzheimer’s disease’.

A gene was assigned to each lead variant—the protein-coding gene in which the lead variant is located, and, otherwise, the nearest protein-coding gene according to Variant Effect Predictor (VEP) (release 109)^31^ and considering only transcripts with the GENCODE basic tag. The locus was named according to the gene assigned to its lead variant and to the locus name in the literature for known loci.

The ideogram was generated using PhenoGram (https://ritchielab.org/software/phenogram-downloads).

### Conditional and joint analyses

To identify secondary signals independent of the lead variant signal in the loci, a stepwise conditional analysis was performed in each locus, except *APOE*, with GCTA COJO^32,33^ based on the summary statistics of the main meta-analysis, and on the LD computed in the EADB-core samples. For this purpose, EADB-core genetic data were converted to best-guess genotype data using a genotype probability threshold of 0.8. Only variants analyzed in ≥70% effective number of cases were considered, leading to the exclusion of the *ADGRL3* locus from these analyses. The *P*-value threshold for defining secondary signals was set at 1 × 10^−5^. Then, to check the independence of the signals across loci, a joint analysis was performed using GCTA COJO of (1) the 157 index variants of the lead and secondary signals detected by the stepwise conditional analysis (Supplementary Note) and (2) the *ADGRL3* lead variant. These conditional and joint analyses were also performed for the no-proxy and no-biobank sensitivity meta-analyses. To assess the sensitivity of the results of the approximate stepwise conditional analysis to LD and imputation quality, we performed (1) a strict stepwise conditional analysis and (2) an exact conditional analysis. The strict stepwise conditional analysis was performed on variants with imputation quality >0.8 in the EADB-core dataset and analyzed in ≥90% effective number of cases. Exact conditional analyses were performed using SNPTEST^34,35^ on raw data from a subset of studies—(1) between the index variants of the lead and secondary signals within the same locus; and (2) between the index variants of the main and secondary signals at the *KAT8*/*BCKDK* and *DOC2A* loci on the one hand and the *APH1B* and *SNX1*/*CIAO2A* loci on the other hand (Supplementary Note). The following studies were considered: EADB-core, Bonn, DemGene, EADI, GERAD, Gothenburg, STSA, TwinGene and all the case–control ADGC studies. Exact conditional results were combined across studies using an inverse-variance-weighted approach, as implemented in METAL.

To compare signals across the main, no-proxy and no-biobank meta-analyses, we performed two joint analyses using GCTA COJO. We jointly analyzed all index variants of the lead and secondary signals from the following: (1) the main and no-biobank meta-analyses and (2) the main and no-proxy meta-analyses. Analyses were restricted to loci with a secondary signal in at least one of the two meta-analyses being compared, while all variants were jointly tested across those loci. Both joint analyses were performed on the summary statistics of the main meta-analysis. From each joint analysis, we excluded one index variant from each pair in LD (*r*^2^ > 0.75 in the EADB-core dataset, as computed by PLINK 2.0) to avoid collinearity. In each such pair, the index variant from the main meta-analysis was retained.

### Rare-variant analysis

We extracted 65 protein-coding genes with the GENCODE basic tag (v43) located within the 16 new tier 1 loci, based on the start and end positions of each locus. Of these, 51 genes were available in the ADES-ADSP summary statistics for the comparison of gene-based rare-variant burdens between 12,652 AD cases and 8,693 controls^10^ (Supplementary Table 8). These samples largely overlap with the ones included in the meta-analysis. We considered a significance threshold of *P* < 9.8 × 10^−4^, corresponding to a Bonferroni correction for 51 tests.

### Single-cell enrichment analysis

We assessed the association between gene overexpression in specific cell types relative to average gene expression and gene associations with ADRD risk using the three-step process implemented in FUMA (v1.6.1)^36^. As input, we used the MAGMA gene-level results provided for the pathway analysis. We tested six models—one primary model and five sensitivity models—corresponding to the primary, common-only, no-APOE, larger-window, no-proxy and no-biobank gene-level summary statistics. We used gene expression data from six datasets of adult human brain tissue—GSE168408_Human_Prefrontal_Cortex_level2_Adult^37^, GSE168408_Human_Prefrontal_Cortex_level1_Adult^37^, PsychENCODE_Adult^38^, DroNc_Human_Hippocampus^39^, Allen_Human_MTG_level1 (middle temporal gyrus) and Allen_Human_MTG_level2 (ref. ^40^). The FUMA three-step process is further described in the Supplementary Note.

### Pathway analyses

Pathway analyses were performed using MAGMA (v1.08)^41,42^, with correction for the number of variants in each gene, LD between variants and LD between genes. LD was computed from the EADB-core dataset using high-quality imputed genotypes (imputation quality of >0.8) and setting as missing genotypes with genotype probability of <0.9. The measure of pathway enrichment was the MAGMA ‘competitive’ test (in which the association statistic for genes in the pathway is compared with those for all other protein-coding genes), as recommended in ref. ^43^. We applied the ‘mean’ test statistic, which sums the −log(*variant P*) across all genes. The total sample size (*n*) was used. A total of 8,034 gene sets were considered for analysis (Supplementary Note). Eight pathway analyses were performed using results from the following: (1) the main meta-analysis (‘primary’ model); (2) the main meta-analysis restricted to common variants (MAF > 0.01; ‘common-only’ model); (3) the main meta-analysis after excluding the *APOE* region (44–46 Mb on chromosome 19 in GRCh38; ‘no-APOE’ model); (4) the main meta-analysis, but mapping variants to genes using a 35-kb upstream and 10-kb downstream window (‘larger-window’ model); (5) the no-proxy meta-analysis (‘no-proxy’ model); (6) the no-proxy meta-analysis restricted to common variants (‘common-only no-proxy’ model); (7) the no-biobank meta-analysis (‘no-biobank’ model) and (8) the no-biobank meta-analysis restricted to common variants (‘common-only no-biobank’ model).

### Phenome-wide association study

Using FUMA (v1.5.2)^44^, we extracted all variants in LD (*r*^2^ > 0.75) with the index variants of the new tier 1 and tier 2 loci in the EUR population from 1000 Genomes Phase 3. The index variant rs7481951 was not available in FUMA and was replaced by rs10833712, its best tag variant (*r*^2^ = 0.827) according to TopLD^45^. We then extracted from the GWAS Catalog (e112_r2024-07-08) all traits associated with these variants at the GWS level.

We also extracted the results for the frequent (MAF > 1%) index variants of the tier 1 and tier 2 loci from the GWAS of 11 NPEs^17^. The samples included in the NPE GWAS largely overlap with the ones included in the ADRD meta-analysis.

### Genetic correlation analyses

Using the no-proxy ADRD GWAS summary statistics, we computed with LDSC (v1.0.1)^21,46^ the genetic correlation between ADRD and Parkinson’s disease^47^, frontotemporal dementia^48^, frontotemporal lobar degeneration with neuronal inclusions of TAR DNA-binding protein 43 (ref. ^49^), Lewy body dementia^50^, amyotrophic lateral sclerosis^51^, educational attainment^52^, stroke and its subtypes^53^ and 11 NPEs^17^. For amyotrophic lateral sclerosis, frontotemporal lobar degeneration with neuronal inclusions of TAR DNA-binding protein 43, Lewy body dementia, Parkinson’s disease and the stroke phenotypes, we used the harmonized version of the summary statistics available in the GWAS Catalog^54^. We used the precomputed ‘eur_w_ld_chr’ LD scores derived from 1000 Genomes European data. The analysis was restricted to HapMap 3 variants and excluded variants in the *APOE* locus, A/T or C/G alleles, variants with MAF of <1%, with duplicated rsID and indels. Correlation was considered significant at *P* < 3.13 × 10^−3^, corresponding to a Bonferroni correction for the 16 phenotypes for which genetic correlation could be computed. The no-proxy summary statistics were selected for this analysis to maximize power while avoiding biases that can arise when using genome-wide summary statistics from studies including proxy cases^14–16^. However, we assessed the sensitivity of the results using the main and no-biobank ADRD summary statistics.

### PGS analyses

We constructed three PGSs using the tier 1 main and secondary signals (except *APOE*) detected in the main, no-proxy and no-biobank meta-analyses, respectively (Supplementary Table 15), and tested their association with the 11 NPEs in the ADC/NACC and ACT studies. Ordinal NPEs (amyloid-β plaques, CERAD score, arteriolosclerosis, atherosclerosis, cerebral amyloid angiopathy, LATE-NC and Lewy body) were dichotomized in two groups (none/mild versus moderate/severe), as well as Braak neurofibrillary tangle stage (stages 0–3 versus stages 4–6). We first considered the GWS lead and secondary tier 1 signals detected in the main analysis as candidate signals. For each PGS (main, no-proxy and no-biobank), we then (1) selected only the candidate signals that were GWS in the respective meta-analysis and (2) selected the index variant of each of those signals in the respective meta-analysis. A total of 115, 91 and 65 variants were finally considered to compute the main, no-proxy and no-biobank PGS, respectively (Supplementary Note). These scores were computed for each individual with PLINK 2.0 using the function ‘score’ as the weighted average of the number of risk-increasing alleles for each variant, using dosages, and were scaled to obtain the PGS^55^:

$$\mathrm{PGS}=\frac{n}{{\sum }_{i=1}^{n}\log \left({\mathrm{OR}}_{i}\right)}\times \mathop{\sum }\limits_{i=1}^{n}\log \left({\mathrm{OR}}_{i}\right)\times {d}_{i}$$

where *n* is the number of variants included in the score, and OR_*i*_ and *d*_*i*_ are the OR and dosage, respectively, of the risk allele of variant *i*. Each OR represents the impact of the variant on ADRD risk and was estimated by repeating the meta-analysis after excluding the biobanks (UKBB, FinnGen, deCODE and HUNT) and the studies overlapping with the NPE datasets (ACT, ADC/NACC, ROSMAP1 and CSDC). ORs were then estimated with GCTA COJO by performing for each score separately a joint analysis of all variants included, following the same pipeline as in the conditional and joint analyses described above (Supplementary Table 15). The effect size estimated for chr4:993555:G:T was considered for the tag variant chr4:973547:G:T. Associations between each binary NPE and each PGS were measured using logistic regression in the ADC/NACC and ACT studies separately. Models were adjusted for age at death, sex, number of *APOE* ε4 and ε2 alleles, ten PCs and centers. Effect sizes were then meta-analyzed across studies in METAL using a fixed-effects inverse-variance weighted approach. An association was considered significant if the *P* value was <2.27 × 10^−3^, corresponding to a Bonferroni correction for 22 tests (11 NPEs analyzed in two studies). Sensitivity analyses were conducted by additionally adjusting for the following: (1) AD diagnosis, coded in three categories (not impaired, AD/mild cognitive impairment and unknown/other dementia); (2) the three AD NPEs; (3) both AD diagnosis and the three AD NPEs or (4) AD NPEs and LATE-NC. For the Braak stage and CERAD score, we additionally tested the interaction between PGS and the number of *APOE* ε4 and ε2 alleles.

The OR for the association with the PGS measures the effect of carrying one additional average-risk allele. These ORs cannot be compared across the three scores because the average risk differs for each. To allow comparisons across scores, we divided each genetic score into quintiles and deciles based on the pooled distribution of PGS across all individuals from the ADC/NACC and ACT studies. We then computed in ADC/NACC the association of Braak stage and CERAD score with PGS deciles using the same model as the raw genetic scores, with the median quintile as the reference group. To allow comparison with the smaller ACT study, association results were also computed per quintiles in both ACT and ADC/NACC.

The discriminative performance of the PGS was assessed through three statistics as follows: (1) the AUC; (2) Nagelkerke’s pseudo-*R*^2^ (ref. ^56^) and (3) *R*^2^ on the liability scale. AUC was computed for each study separately with the ‘auc’ function from the pROC (v1.18.5) R package^57^, for both the null logistic model, including only age, sex, *APOE* ε4 and ε2, PCs and centers, and the full model, which additionally included the PGS. The AUCs between the two models were then compared with the DeLong’s test^58^ as implemented in the ‘roc.test’ function. The same pipeline was applied to compare AUCs across models, including the main, no-proxy and no-biobank PGSs. Nagelkerke pseudo-*R*^2^ was computed with the ‘nagelkerke’ function from the R package rcompanion (v2.5.0)^59^. We also computed the variance explained by each PGS on the observed scale as the difference in the fraction of variance explained by a linear model under the full and null models. It was then transformed to the liability scale using the approach as described in ref. ^60^, with population prevalence values ranging from 0.1 to 0.9.

### Reporting summary

Further information on research design is available in the Nature Portfolio Reporting Summary linked to this article.

## Online content

Any methods, additional references, Nature Portfolio reporting summaries, source data, extended data, supplementary information, acknowledgements, peer review information; details of author contributions and competing interests; and statements of data and code availability are available at 10.1038/s41588-026-02583-1.

## Supplementary information

Supplementary Information Supplementary Note and Supplementary Figs. 1–68.

Reporting Summary

Peer Review File

Supplementary Tables Supplementary Tables 1–25.

## Data Availability

Summary statistics of the main, no-proxy and no-biobank meta-analyses are available through the European Bioinformatics Institute GWAS Catalog (https://www.ebi.ac.uk/gwas/) with accessions GCST90704646, GCST90704647 and GCST90704648 and through NIAGADS (https://dss.niagads.org/). Genetic scores are available in Supplementary Table 15 and through the PGS Catalog (https://www.pgscatalog.org/) with accessions PGS005389, PGS005390 and PGS005391.

## Code availability

The software we used is referenced in the Methods and Supplementary Note, and the corresponding URLs are provided in the Supplementary Note. Additional scripts are available on Zenodo (10.5281/zenodo.18324799)^61^.
