# Supplementary material for: Consensus meta-analysis of genome-wide association studies for Alzheimer’s disease and related dementias
Source: Nat Genet. 2026 Jun 3;58(6):1214–25. doi: 10.1038/s41588-026-02583-1 (PMC13263136; doi:10.1038/s41588-026-02583-1)
Supplement: Supplementary file 2 — Reporting Summary [file 41588_2026_2583_MOESM2_ESM.pdf]

nature portfolio

Corresponding author(s): Céline Bellenguez
Jean-Charles Lambert

Last updated by author(s): 2026/01/20

Reporting Summary

Nature Portfolio wishes to improve the reproducibility of the work that we publish. This form provides structure for consistency and transparency in reporting. For further information on Nature Portfolio policies, see our Editorial Policies and the Editorial Policy Checklist.

Statistics

For all statistical analyses, confirm that the following items are present in the figure legend, table legend, main text, or Methods section.

n/a	Confirmed	
☐	☒	The exact sample size (n) for each experimental group/condition, given as a discrete number and unit of measurement
☒	☐	A statement on whether measurements were taken from distinct samples or whether the same sample was measured repeatedly
☐	☒	The statistical test(s) used AND whether they are one- or two-sided<br>*Only common tests should be described solely by name; describe more complex techniques in the Methods section.*
☐	☒	A description of all covariates tested
☐	☒	A description of any assumptions or corrections, such as tests of normality and adjustment for multiple comparisons
☐	☒	A full description of the statistical parameters including central tendency (e.g. means) or other basic estimates (e.g. regression coefficient) AND variation (e.g. standard deviation) or associated estimates of uncertainty (e.g. confidence intervals)
☐	☒	For null hypothesis testing, the test statistic (e.g. F, t, r) with confidence intervals, effect sizes, degrees of freedom and P value noted<br>*Give P values as exact values whenever suitable.*
☒	☐	For Bayesian analysis, information on the choice of priors and Markov chain Monte Carlo settings
☒	☐	For hierarchical and complex designs, identification of the appropriate level for tests and full reporting of outcomes
☐	☒	Estimates of effect sizes (e.g. Cohen's d, Pearson's r), indicating how they were calculated

Our web collection on statistics for biologists contains articles on many of the points above.

Software and code

Policy information about availability of computer code

Data collection

No software was used for data collection

Data analysis

The software we used is referenced in the Online Methods and Supplementary Information, and the corresponding URLs are provided in the Supplementary Information
Bedtools: https://bedtools.readthedocs.io
BCFtools: http://samtools.github.io/bcftools/bcftools.html
Gene Ontology (downloaded from the NCBI on June 19th, 2023): http://geneontology.org/docs/download-ontology/
Reactome (July 17th, 2023): https://reactome.org/download-data
KEGG and Pathway Interaction Database (PID) pathways (MSigDB v2023.1.Hs updated March 2023): https://www.gsea-msigdb.org/gsea/msigdb/index.jsp
LocusZoom: https://github.com/statgen/locuszoom-standalone
Sanger imputation server: https://imputation.sanger.ac.uk/
Michigan imputation server: https://imputationserver.sph.umich.edu/
SNPTEST 2.5.4-beta1, 2.5.4-beta3, and 2.5.6: https://www.chg.ox.ac.uk/~gav/snptest/
FlashPCA2: https://github.com/gabraham/flashpca
EIGENSOFT: https://www.hsph.harvard.edu/alkes-price/software/
PLINK 1.9: https://www.cog-genomics.org/plink/
PLINK 2.0: https://www.cog-genomics.org/plink/2.0/
GMMAT 1.4.2: https://cran.r-project.org/web/packages/GMMAT/index.html
SAIGE v.0.35.8.3 and 1.0.9: https://saigegit.github.io/SAIGE-doc/
regenie v2.2.4: https://rgcgithub.github.io/regenie/

GCTA-COJO: https://yanglab.westlake.edu.cn/software/gcta/#COJO
FUMA v1.5.2 and v1.6.1: https://fuma.ctglab.nl/
UCSC LiftOver: https://genome.ucsc.edu/cgi-bin/hgLiftOver
McCarthy group tools: https://www.well.ox.ac.uk/~wrayner/tools/
Human Genome Diversity Project (HGDP) reference panel: http://csg.sph.umich.edu/chaolong/LASER/
Minimac4: https://genome.sph.umich.edu/wiki/Minimac4
LD Score Regression: https://github.com/bulik/ldsc
METAL v2020-05-05: https://github.com/statgen/METAL/tree/master
LDstore 2: http://www.christianbenner.com/
emeraLD: http://github.com/statgen/emeraLD
GWAS catalog version e112_r2024-07-08: https://www.ebi.ac.uk/gwas/
VEP: https://www.ensembl.org/info/docs/tools/vep/index.html
PhenoGram: https://ritchielab.org/software/phenogram-downloads
ADES-ADSP summary statistics: https://doi.org/10.5281/zenodo.6818051
MAGMA v1.08: https://cncr.nl/research/magma/
ontologyIndex R package: https://cran.r-project.org/web/packages/ontologyIndex/index.html
qqman R package: https://cran.r-project.org/web/packages/qqman/index.html
GenABEL R package (estlambda function): https://github.com/GenABEL-Project/GenABEL
rmeta R package (forestplot function): https://cran.r-project.org/web/packages/rmeta/index.html
ggplot2 R package: https://cran.r-project.org/web/packages/ggplot2/index.html
1000 Genomes data, Phase 3: https://www.internationalgenome.org/data
pROC R package: https://cran.r-project.org/web/packages/pROC/index.html
rcompanion R package: https://cran.r-project.org/web/packages/rcompanion/index.html

Additional scripts are available through Zenodo (https://doi.org/10.5281/zenodo.18324799).

For manuscripts utilizing custom algorithms or software that are central to the research but not yet described in published literature, software must be made available to editors and reviewers. We strongly encourage code deposition in a community repository (e.g. GitHub). See the Nature Portfolio guidelines for submitting code & software for further information.

Data

Policy information about availability of data

All manuscripts must include a data availability statement. This statement should provide the following information, where applicable:

- Accession codes, unique identifiers, or web links for publicly available datasets
- A description of any restrictions on data availability
- For clinical datasets or third party data, please ensure that the statement adheres to our policy

Summary statistics of the main, no-proxy and no-biobank meta-analyses are available through the European Bioinformatics Institute GWAS Catalog (https://www.ebi.ac.uk/gwas/) under accessions GCST90704646, GCST90704647 and GCST90704648 and through NIAGADS (https://dss.niagads.org/). Genetic scores are available in Supplementary Table 15 and through the PGS catalog (https://www.pgscatalog.org/) under accessions PGS005389, PGS005390 and PGS005391.

Research involving human participants, their data, or biological material

Policy information about studies with human participants or human data. See also policy information about sex, gender (identity/presentation), and sexual orientation and race, ethnicity and racism.

Reporting on sex and gender	Concordance check between clinical and genetic sex was performed.<br>Statistics on sex are reported in Supplementary Tables 1 and 16<br>Main GWAS analyses were not adjusted on sex but Supplementary Table 24 provides results after adjustment on sex for the index variants detected in the main meta-analysis
Reporting on race, ethnicity, or other socially relevant groupings	The samples were of European ancestry, and the analyses were adjusted on principal components measuring ancestry. The methods used to define ancestry differ for each study (principal component analyses on genetic data, self-reported ancestry...). Deatils are provided for each study in the Supplementary Materials or in the provided references.
Population characteristics	Summary statistics on age, sex and APOE status are provided for each study in Supplementary Tables 1 and 16.
Recruitment	Individuals were recruited from a lot of different centers, with the different recruitement strategies detailed in the Supplementary Material. This includes recruitment from clinics, nursing homes, disease registries and hospital, but also adult volunteers. Cases were recruited according to clinical diagnosis and defined as probable AD cases with a potential risk of mis-diagnosis (estimated between 10 and 20% in the litterature). A large part of the controls did not have any follow-up, and not all of them were screened for dementia (for example in biobanks) so some may have AD or may develop AD later. In biobanks such as the UK biobank, participants are healthier than in the general population. Additionally, the use of proxy-cases can lead to some biases in genetic analyses performed on genome-wide summary statistics. Sensitivity analyses were thus conducted after exclusion of proxy and biobank samples.
Ethics oversight	The appropriate review boards from the ADGC, Bonn, CHARGE, EADB, EADI, GERAD, GR@ACE/DEGESCO and PGC-ALZ reviewed and approved the study protocol.

Note that full information on the approval of the study protocol must also be provided in the manuscript.

Field-specific reporting

Please select the one below that is the best fit for your research. If you are not sure, read the appropriate sections before making your selection.

☒ Life sciences ☐ Behavioural & social sciences ☐ Ecological, evolutionary & environmental sciences

For a reference copy of the document with all sections, see nature.com/documents/nr-reporting-summary-flat.pdf

Life sciences study design

All studies must disclose on these points even when the disclosure is negative.

Sample size	No sample-size calculation was performed, we maximized the number of European-ancestry samples with genotyping data and AD status
Data exclusions	A standard quality control of the genetic data was performed in each study, leading to the exclusion of some variants ans some samples. The quality control is detailed in the Supplementary Material or references are provided.
Replication	We did not attempt to replicate the new hits in an independent sample, as we included all the studies in the discovery step. Sensitivity analyses were performed by excluding proxy-samples or biobank samples. Forest plots are provided to assess heterogeneity of the hit results across studies.
Randomization	There was no allocation in experimental groups as we used ADRD (proxy-) cases and controls from retrospective studies.
Blinding	Investigators were blinded during the genotyping process but not during the quality controls, as some of the quality controls are based on the case/control status

Reporting for specific materials, systems and methods

We require information from authors about some types of materials, experimental systems and methods used in many studies. Here, indicate whether each material, system or method listed is relevant to your study. If you are not sure if a list item applies to your research, read the appropriate section before selecting a response.

Materials & experimental systems

n/a	Involved in the study	
☒	☐	Antibodies
☒	☐	Eukaryotic cell lines
☒	☐	Palaeontology and archaeology
☒	☐	Animals and other organisms
☒	☐	Clinical data
☒	☐	Dual use research of concern
☒	☐	Plants

Methods

n/a	Involved in the study	
☒	☐	ChIP-seq
☒	☐	Flow cytometry
☒	☐	MRI-based neuroimaging

Plants

Seed stocks	*Report on the source of all seed stocks or other plant material used. If applicable, state the seed stock centre and catalogue number. If plant specimens were collected from the field, describe the collection location, date and sampling procedures.*
Novel plant genotypes	*Describe the methods by which all novel plant genotypes were produced. This includes those generated by transgenic approaches, gene editing, chemical/radiation-based mutagenesis and hybridization. For transgenic lines, describe the transformation method, the number of independent lines analyzed and the generation upon which experiments were performed. For gene-edited lines, describe the editor used, the endogenous sequence targeted for editing, the targeting guide RNA sequence (if applicable) and how the editor was applied.*
Authentication	*Describe any authentication procedures for each seed stock used or novel genotype generated. Describe any experiments used to assess the effect of a mutation and, where applicable, how potential secondary effects (e.g. second site T-DNA insertions, mosiacism, off-target gene editing) were examined.*
