# Supplementary material for: Consensus meta-analysis of genome-wide association studies for Alzheimer’s disease and related dementias
Source: Nat Genet. 2026 Jun 3;58(6):1214–25. doi: 10.1038/s41588-026-02583-1 (PMC13263136; doi:10.1038/s41588-026-02583-1)
Supplement: Supplementary file 3 — Peer Review File [file 41588_2026_2583_MOESM3_ESM.pdf]

nature portfolio

Peer Review File

Consensus meta-analysis of genome-wide association studies for Alzheimer’s disease and related dementias

Corresponding Author: Dr Céline Bellenguez

This file contains all editorial decision letters in order by version, followed by all author rebuttals in order by version.

Version 0:

Decision Letter:

7th March 2025

Dear Céline,

Your Brief Communication "Consensus meta-analysis of genome-wide association studies for Alzheimer’s disease and related dementia" has been seen by two referees. You will see from their comments below that, while they find your work of interest, they have raised several relevant points. We are interested in the possibility of publishing your study in Nature Genetics, but we would like to consider your response to these points in the form of a revised manuscript before we make a final decision on publication.

To guide the scope of the revisions, the editors discuss the referee reports in detail within the team, including with the chief editor, with a view to identifying key priorities that should be addressed in revision, and sometimes overruling referee requests that are deemed beyond the scope of the current study. In this case, we ask that you to revise the presentation to better place this work in context with previous studies, extend the analyses where feasible as suggested by Reviewer #2, and reformat the manuscript as a Letter to accommodate these additional analyses and discussion. We hope that you will find this prioritized set of referee points to be useful when revising your study. Please do not hesitate to get in touch if you would like to discuss these issues further.

We therefore invite you to revise your manuscript taking into account all reviewer and editor comments. Please highlight all changes in the manuscript text file. At this stage, we will need you to upload a copy of the manuscript in MS Word .docx or similar editable format.

We are committed to providing a fair and constructive peer-review process. Do not hesitate to contact us if there are specific requests from the reviewers that you believe are technically impossible or unlikely to yield a meaningful outcome.

When revising your manuscript:

*1) Include a “Response to referees” document detailing, point-by-point, how you addressed each referee comment. If no action was taken to address a point, you must provide a compelling argument. This response will be sent back to the referees along with the revised manuscript.

*2) If you have not done so already, please begin to revise your manuscript so that it conforms to our formatting instructions, available
here.
Refer also to any guidelines provided in this letter.

*3) Include a revised version of any required Reporting Summary: https://www.nature.com/documents/nr-reporting-summary.pdf

It will be available to referees (and, potentially, statisticians) to aid in their evaluation if the manuscript goes back for peer review. A revised checklist is essential for re-review of the paper.

Please be aware of our guidelines on digital image standards.

EXTENDED DATA FIGURES

When re-submitting your manuscript, please ensure that any supplementary figures and tables that are crucial to the manuscript's conclusions are converted into Extended Data figures and tables to increase visibility of these data. Extended Data figures and tables are online-only (present in the online PDF and full-text HTML versions of the paper), peer-reviewed display items that provide essential background to the article but are not included in the main article due to space constraints. A maximum of ten Extended Data display items (figures and tables) is permitted.

Please use the link below to submit your revised manuscript and related files:

Link Redacted

Note: This URL links to your confidential home page and associated information about manuscripts you may have submitted, or that you are reviewing for us. If you wish to forward this email to co-authors, please delete the link to your homepage.

We hope to receive your revised manuscript within 4-8 weeks. If you cannot send it within this time, please let us know.

Please do not hesitate to contact me if you have any questions or would like to discuss these revisions further.

Nature Genetics is committed to improving transparency in authorship. As part of our efforts in this direction, we are now requesting that all authors identified as 'corresponding author' on published papers create and link their Open Researcher and Contributor Identifier (ORCID) with their account on the Manuscript Tracking System (MTS), prior to acceptance. ORCID helps the scientific community achieve unambiguous attribution of all scholarly contributions. You can create and link your ORCID from the home page of the MTS by clicking on 'Modify my Springer Nature account'. For more information, please visit www.springernature.com/orcid.

We look forward to seeing the revised manuscript and thank you for the opportunity to review your work.

Sincerely,
Kyle

Kyle Vogan, PhD
Senior Editor
Nature Genetics
https://orcid.org/0000-0001-9565-9665

Referee expertise:

Referee #1: Genetics, neurodegenerative diseases

Referee #2: Genetics, neurodegenerative diseases

Reviewers' Comments:

Reviewer #1 (Remarks to the Author):

The brief communication presents updated results from GWAS meta-analysis of ADRD. The sample size has been further expanded, and the number of identified loci has also increased. The paper emphasizes the conditional results and the findings from the sensitivity analysis conducted after excluding proxy cases. Classical post-analysis was performed to validate the primary findings and provide biological insights. I only have minor suggestions:

1. More clear descriptions of the definitions of tier 1 and tier 2 loci are needed, either in the main text or in the figure legend, to enhance reader understanding.

2. The criteria for what constitutes "validated" as mentioned in line 49 is unclear and should be elaborated.

3. When comparing this study with existing AD GWAS research, the level of innovation appears insufficient. It would be beneficial to discuss the unique contributions of this study beyond the identification of additional loci.

Reviewer #2 (Remarks to the Author):

This manuscript is a timely, much-needed consensus meta-analysis of genome-wide association studies for Alzheimer's disease (AD) and related dementia. It brings together three different consortia, after several publications in recent years with overlapping samples, but varying sample sizes and use of biobanks such as UK Biobank including AD by proxy cases (parental diagnosis of dementia). The large sample size includes a total of 72,721 cases, 55,960 proxy cases and 614,267 controls and 235,566 proxy controls. This is the largest GWAS meta-analysis to date identifying novel loci. 91 genome-wide significant loci were identified (defined as Tier 1). In addition, 5 loci were identified where the significance level dropped below genome-wide significance after conditional analysis (defined as Tier 2).

To further delineate the impact of known genomic loci on AD vs ADRD, sensitivity analyses were performed by excluding proxy or large biobank samples. It needs to be made clearer why this sensitivity analysis is required by referring more to the bias that may be introduced by the use of proxy AD cases and large biobank samples (for example, reference Wu et al. Nat. Genet. 2024 Dec;56(12):2696-2703, Escott-Price, Hardy J, Brain Commun. 2022 May 17;4(3)). This is important to allow readers to make an informed choice about which of the provided summary stats they should use for future work, such as Mendelian Randomisation analysis.

Downstream analysis included pathway analysis using MAGMA, a phenome-wide association study, and an investigation of gene expression in specific cell types in the brain.

Although this paper is in a brief communication format, there appear to be some key analyses missing. There is no comparison of results with previous GWAS meta-analyses aside from the reporting of novel variants. The SNP-based heritability is not reported. There is no test of polygenic scores to test the predictive utility of the findings, or genetic correlations reported with other traits.

Version 1:

Decision Letter:

Our ref: NG-LE67541R

13th November 2025

Dear Céline,

Your revised manuscript "Consensus meta-analysis of genome-wide association studies for Alzheimer's disease and related dementia" (NG-LE67541R) has been seen by the original reviewers. As you will see from their comments below, they are satisfied with your responses to their previous comments, and therefore we will be happy in principle to publish your study in Nature Genetics as a Letter pending final revisions to satisfy Reviewer #2's remaining request and to comply with our editorial and formatting guidelines.

We are now performing detailed checks on your paper, and we will send you a checklist detailing our editorial and formatting requirements soon. Please do not upload the final materials or make any revisions until you receive this additional information from us.

Thank you again for your interest in Nature Genetics. Please do not hesitate to contact me if you have any questions.

Sincerely,
Kyle

Kyle Vogan, PhD
Senior Editor
Nature Genetics
https://orcid.org/0000-0001-9565-9665

Reviewer #1 (Remarks to the Author):

The authors have adequately addressed all my previous comments, and I have no further remarks.

Reviewer #2 (Remarks to the Author):

Thank you for the clear response to all the reviewer's comments and the changes made. All the concerns have been addressed.

My only additional comment is concerning the recent preprint publication of the preprint Uffelmann et al. (https://www.medrxiv.org/content/10.1101/2025.10.10.25337470v1) which was recently made available on the 13/10/25. As I

understand this would be the largest multi-ancestry AD GWAS now available, for completeness it would be useful for this to be referred to in the manuscript, in the same way as the other large scale GWAS studies, detailing any overlaps/replications.

Open Access This Peer Review File is licensed under a Creative Commons Attribution 4.0 International License, which permits use, sharing, adaptation, distribution and reproduction in any medium or format, as long as you give appropriate credit to the original author(s) and the source, provide a link to the Creative Commons license, and indicate if changes were made.
In cases where reviewers are anonymous, credit should be given to 'Anonymous Referee' and the source.
The images or other third party material in this Peer Review File are included in the article's Creative Commons license, unless indicated otherwise in a credit line to the material. If material is not included in the article's Creative Commons license and your intended use is not permitted by statutory regulation or exceeds the permitted use, you will need to obtain permission directly from the copyright holder.
To view a copy of this license, visit https://creativecommons.org/licenses/by/4.0/

Reviewer #1:

The brief communication presents updated results from GWAS meta-analysis of ADRD. The sample size has been further expanded, and the number of identified loci has also increased. The paper emphasizes the conditional results and the findings from the sensitivity analysis conducted after excluding proxy cases. Classical post-analysis was performed to validate the primary findings and provide biological insights. I only have minor suggestions:

1. More clear descriptions of the definitions of tier 1 and tier 2 loci are needed, either in the main text or in the figure legend, to enhance reader understanding.

A clear definition of tier 1 and tier 2 loci is now provided in the legend of Figure 1: « Tier 1 loci are considered genuine genome-wide significant signals, with $P \leq 5x10^{-8}$ in the unconditional analysis and $P \leq 1x10^{-7}$ in conditional analyses within or across loci. Tier 2 loci are genome-wide significant signals requiring further external validation because i) P was above $1x10^{-7}$ after conditional analyses or ii) they were genome-wide significant only in the sensitivity no-proxy and no-biobank meta-analyses. »

2. The criteria for what constitutes "validated" as mentioned in line 49 is unclear and should be elaborated.

We made it more clear by replacing the word « validated » by « genome-wide significant » (now line 55).

3. When comparing this study with existing AD GWAS research, the level of innovation appears insufficient. It would be beneficial to discuss the unique contributions of this study beyond the identification of additional loci.

The main contribution of this GWAS is indeed the identification of 16 new loci and 15 new loci requiring further validation.

In addition, this study allowed to further characterized the impact of known loci by

i) validating – or not – their association with ADRD risk : *HAVCR2*, *SLC2A4RG/LIME1*, *FOXF1* and *NTN5* were not validated (lines 50-51) while *SEC61G/EGFR*, *SPPL2A/USP8/USP50* and *KAT8/BCKDK* require further validation (lines 45-47)

ii) identifying new secondary signals in some of them (*CD33*, *HLA*, *PICALM*, *RHOH*, *ABCA7*, *BIN1*, *PTK2B/CLU*, *NCK2* and *PLCG2*, lines 40-41) ;

iii) validating their association in a larger AD case-control sample excluding proxy or large biobank samples. We now highlight that « 12 known loci were genome-wide significant for the first time after focusing on clinically diagnosed AD cases (*NCK2*, *MME*, *ANKH/OTULIN*, *ABCA1*, *BLNK*, *IGH*, *ATP8B4*, *SNX1/CIAO2A*, *IL34/MTSS2*, *SIGLEC11*, *RBCK1* and *APP*) » (lines 59-61)

Those contributions are now more clearly stated in the conclusion, lines 138-140: « We also further characterized the impact of known loci by validating – or not – their association with ADRD and AD risk in larger samples and by identifying new secondary signals in some of them. »

We also provide 3 sets of genome-wide summary statistics that can be used for different purposes for secondary genetic analyses. We now highlight this in the conclusion, lines 142-145 « However, assessing the sensitivity of results of GWAS or GWAS secondary analyses (especially those based on genome-wide statistics[14]) on clinically-diagnosed cases will become increasingly important as the proportion of proxy and biobank cases included in the GWAS increases. This will be made easier by the release of the no-proxy and no-biobank summary statistics. »

Finally, we now provide an extensive evaluation of the impact of different ADRD polygenic scores on 11 AD and non-AD neuropathological endophenotypes (lines 93-135) showing that a polygenic score combining the effect of the ADRD loci was primarily associated with AD rather than non-AD pathology.

Reviewer #2 (Remarks to the Author) :

This manuscript is a timely, much-needed consensus meta-analysis of genome-wide association studies for Alzheimer's disease (AD) and related dementia.

We thank the reviewers for their positive feedback on the paper.

It brings together three different consortia, after several publications in recent years with overlapping samples, but varying sample sizes and use of biobanks such as UK Biobank including AD by proxy cases (parental diagnosis of dementia). The large sample size includes a total of 72,721 cases, 55,960 proxy cases and 614,267 controls and 235,566 proxy controls. This is the largest GWAS meta-analysis to date identifying novel loci. 91 genome-wide significant loci were identified (defined as Tier 1). In addition, 5 loci were identified where the significance level dropped below genome-wide significance after conditional analysis (defined as Tier 2).

To further delineate the impact of known genomic loci on AD vs ADRD, sensitivity analyses were performed by excluding proxy or large biobank samples. It needs to be made clearer why this sensitivity analysis is required by referring more to the bias that may be introduced by the use of proxy AD cases and large biobank samples (for example, reference Wu et al. Nat. Genet. 2024 Dec;56(12):2696-2703, Escott-Price, Hardy J, Brain Commun. 2022 May 17;4(3)). This is important to allow readers to make an informed choice about which of the provided summary stats they should use for future work, such as Mendelian Randomisation analysis.

We have now added references to biases that may be introduced by the use of proxy samples when performing GWAS

1) lines 89-92: "Those results, consistent with previous studies[13,14], were similar when considering the main and no-biobank summary statistics except for educational attainment when using the main meta-analysis results (r=-0.02, 95% CI [-0.07, 0.02]). This is in accordance with previous reports of biases observed with genome-wide summary statistics of studies including proxy cases[14–16]."

2) lines 142-145: « However, assessing the sensitivity of results of GWAS or GWAS secondary analyses (especially those based on genome-wide statistics[14]) on clinically-diagnosed cases will become increasingly important as the proportion of proxy and biobank cases included in the GWAS increases. This will be made easier by the release of the no-proxy and no-biobank summary statistics. »

Downstream analysis included pathway analysis using MAGMA, a phenome-wide association study, and an investigation of gene expression in specific cell types in the brain.

Although this paper is in a brief communication format, there appear to be some key analyses missing. There is no comparison of results with previous GWAS meta-analyses aside from the reporting of novel variants.

In addition to the report of 16 new loci, 15 new loci requiring further validation and some new secondary signals in known loci, we highlighted that *HAVCR2*, *SLC2A4RG/LIME1*, *FOXF1* and *NTN5* were not validated (lines 50-51) while *SEC61G/EGFR*, *SPPL2A/USP8/USP50* and *KAT8/BCKDK* require further validation (lines 45-47). In the revision, we now highlight that 12 known loci were genome-wide significant for the first time after excluding large biobanks (*NCK2*, *MME*, *ANKH/OTULIN*, *ABCA1*, *BLNK*, *IGH*, *ATP8B4*, *SNX1/CIAO2A*, *IL34/MTSS2*, *SIGLEC11*, *RBCK1* and *APP*) (lines 59-61). We also stated that the over-expression of ADRD association signals in microglia was previously known (« as in previous GWAS », line 76). We now more clearly state that the pathway results are similar too (« similarly to previous GWAS results », line 79). We now report genetic correlations with other traits, and emphasize that results are consistent with previous reports (line 89).

The SNP-based heritability is not reported.

Heritability is a population-dependent measure and computing the SNP-based heritability on the liability scale using summary statistics, for example with LDSC, requires to provide prevalence of the disease in the population. Now, AD prevalence increases exponentially with age and also varies with sex and the studies included in our meta-analysis have different designs and characteristics. We thus think that our meta-analysis results are not suitable to provide accurate heritability estimations on the liability scale. We however provide the heritability values on the observed scale in Supplementary Table 6 for the sake of completeness.

There is no test of polygenic scores to test the predictive utility of the findings, or genetic correlations reported with other traits.

We have now added analyses of polygenic scores (lines 93-135) and genetic correlations (lines 85-92). We tested the association of ADRD polygenic scores (PGS) with 11 AD and non-AD neuropathological endophenotypes. Our results were consistent across the main, no-proxy and no-biobank PGS and the three PGS were primarily associated with AD rather than non-AD pathology. Genetic correlation results were consistent with those previously reported in the literature.

We hope that our answers and modifications correspond to your expectations as well as those of reviewers.

Looking forward to hearing from you,

Best regards,

Jean-Charles Lambert
Céline Bellenguez

As requested, we provide below a point-by-point answer to the reviewer's comments:

Reviewer #1 (Remarks to the Author):

The authors have adequately addressed all my previous comments, and I have no further remarks.

Reviewer #2 (Remarks to the Author):

Thank you for the clear response to all the reviewer's comments and the changes made. All the concerns have been addressed.

My only additional comment is concerning the recent preprint publication of the preprint Uffelmann et al. (https://www.medrxiv.org/content/10.1101/2025.10.10.25337470v1) which was recently made available on the 13/10/25. As I understand this would be the largest multi-ancestry AD GWAS now available, for completeness it would be useful for this to be referred to in the manuscript, in the same way as the other large scale GWAS studies, detailing any overlaps/replications.

Our paper has been under review for a year, so we are surprised by this request to provide a detailed comparison of our results with those from a very recent unpeer-reviewed preprint. Additionally, this pre-print was made available on the 13th of October, while we were submitting our revised version on the 10th of October according to the manuscript tracking system. We think it would be inappropriate for us to pre-empt the review process, and we believe that commenting on any overlap or difference in results is premature, given that these results may change substantially in the peer-reviewed published version. We also note that the two works differ substantially in terms of sampling and objectives. For instance, approximately 26,000 of our clinically diagnosed AD cases are not included in this preprint which mainly includes new proxy-AD cases and biobank samples. However, one of our objectives was to assess how proxy-AD cases and AD cases in biobanks (not clinically diagnosed) can impact relevance of genetic associations.

Additionally, we modified the paper to meet the formatting requirements. We would like to highlight that some of these modifications are linked to an issue we identified while using the no-proxy summary statistics for another project. As now described in the Methods, "Variants with positions above 80 Mb on chromosome 11 were missing by mistake from the UKBB-diagnosed summary statistics. This issue leads to a small loss of power (6% of the effective sample size) in a small region of the genome

(around 2%) for the no-proxy sensitivity analyses presented in this paper, and has minimal impact on the results" (lines 1244-1248, p 32 of the main text). As further described in the Supplementary Note (p 36-37), Supplementary Figure 65 and Supplementary Table 25, in the corrected no-proxy meta-analysis compared to the no-proxy meta-analysis used in this paper: (i) 10 variants were excluded due to a frequency issue in the UKBB study, (ii) 3,676 variants were added and (iii) 262,719 variants (1.87%) had slightly different results. Indeed, the correlation between the "paper" and "corrected" results were 0.938, 0.999 and 0.984 for the effect sizes, standard errors and p-values respectively (Supplementary Figure 65). Additionally, there are two hits in this region of chromosome 11 (*PICALM* and *SORL1*) and their results are almost the same (Supplementary Table 25). Besides, no additional genome-wide significant signals were detected in the corrected raw no-proxy meta-analysis. As a consequence, we believe the issue should not impact the results using the no-proxy genome-wide summary statistics (pathway analysis, single-cell enrichment analysis, genetic correlation), nor the no-proxy genetic score which used effect sizes estimated after exclusion of UKBB. By the way, the no-proxy meta-analysis is a **sensitivity** analysis and we showed that most results were similar between the three meta-analyses.

For all these reasons, we considered it was not judicious to significantly delay the publication by performing again all the no-proxy analyses. However, as stated in the main text and the Supplementary Note, the corrected summary statistics are provided in the GWAS catalog and on NIAGADS.

Looking forward to hearing from you,

Best regards,

Jean-Charles Lambert
Céline Bellenguez
